# Measuring the bias of incorrect application of feature selection when using cross-validation in radiomics

**DOI:** 10.1186/s13244-021-01115-1

**Published:** 2021-11-24

**Authors:** Aydin Demircioğlu

**Affiliations:** Institute of Diagnostic and Interventional Radiology and Neuroradiology, University Hospital Essen, Hufelandstr. 55, 45147 Essen, Germany

**Keywords:** Radiomics, Feature selection, Cross-validation, Bias, Machine learning

## Abstract

**Background:**

Many studies in radiomics are using feature selection methods to identify the most predictive features. At the same time, they employ cross-validation to estimate the performance of the developed models. However, if the feature selection is performed before the cross-validation, data leakage can occur, and the results can be biased. To measure the extent of this bias, we collected ten publicly available radiomics datasets and conducted two experiments. First, the models were developed by incorrectly applying the feature selection prior to cross-validation. Then, the same experiment was conducted by applying feature selection correctly within cross-validation to each fold. The resulting models were then evaluated against each other in terms of AUC-ROC, AUC-F1, and Accuracy.

**Results:**

Applying the feature selection incorrectly prior to the cross-validation showed a bias of up to 0.15 in AUC-ROC, 0.29 in AUC-F1, and 0.17 in Accuracy.

**Conclusions:**

Incorrect application of feature selection and cross-validation can lead to highly biased results for radiomic datasets.

**Supplementary Information:**

The online version contains supplementary material available at 10.1186/s13244-021-01115-1.

## Key points

- Incorrectly applying feature selection on the whole dataset before cross-validation can cause a large positive bias.
- Datasets with higher dimensionality, i.e., more features per sample, are more prone to positive bias.

## Background

Radiomics is a method to extract and analyze high-dimensional quantitative features from radiological, non-invasive imaging data to enable predictive decision support [1]. The basic assumption of radiomics is that these features correspond to imaging biomarkers that contain characteristic information about diseases. Radiomics potentially allows for patient-centric diagnosis [2] and has been employed for many types of tumors [3–6].

Since it is not known beforehand which feature will be important for the particular outcome considered, radiomics extracts far more features than necessary. Many of these are therefore potentially irrelevant and redundant [7–9]. Thus, various feature selection methods are employed to reduce the features to the most predictive and robust ones, although it is well known that these methods are challenging and can be misleading [10].

Another problem with radiomics is the rather small sample sizes. There are several reasons for this, for example, if the pathology under consideration is rare or suitable data is not readily available. In addition, radiomics often needs segmentations of the pathology, which cannot be performed manually if the sample sizes go into the thousands. Also, access to external data is often restricted because of privacy issues. Therefore, radiomic datasets often comprise only a few hundred samples, which is critical from a statistical viewpoint [11].

Together with the multitude of extracted features, this leads to high-dimensional datasets, i.e. they have fewer samples than features. Since the analysis of such datasets is complex, guidelines and standards were introduced to ensure the validity of radiomics studies [12–15].

A key problem when modeling is overfitting, which occurs when a model learns the noise and peculiarities of a given training dataset rather than the underlying patterns, and therefore does not generalize to new data. Overfitting problems can be identified by using validation data that is not used during training. Since explicit validation data is rarely available, validation schemes are used, where part of the training data is set apart and only used to obtain an unbiased estimate of the performance of the model. Often, cross-validation (CV) is employed, where the data is split into several folds and then used in turn to train and to validate the model.

When cross-validation is employed, clearly all modeling must be applied only to the training folds, else data leakage would occur, which describes the fact that the validation data was already used and estimations could potentially be biased. This is especially true for the feature selection, which is a fundamental part of the radiomics pipeline. Since applying the feature selection before the cross-validation on all data would lead to data leakage, feature selection must be part of the cross-validation for the resulting model to be unbiased and to generalize to new data.

Unfortunately, sometimes illustrations of the radiomics pipeline are simplified and make the impression that feature selection is a preprocessing step before modeling applied to the whole data and that cross-validation is only part of the model selection [3, 12, 16]. Similarly, it is not always clear whether studies that use cross-validation but not an explicit validation set have applied feature selection incorrectly or whether it is just misleadingly described [17–29]. In fact, only a few studies describe their methodology in full [6].

To understand how far incorrect application of feature selection before cross-validation introduces a bias to the analysis, it is important to measure the extent of the difference. Therefore, in this study we utilized 10 radiomics datasets, 7 feature selection methods as well as 7 classifiers and study via a tenfold cross-validation in how far an incorrect order of feature selection and cross-validation has an impact on the estimated performance.

## Methods

All data used in this study were previously published; therefore ethical approval was waived by the local Ethics Committee (Ethik-Kommission, Medizinische Fakultät der Universität Duisburg-Essen, Germany). Methods and procedures were performed in accordance with the relevant guidelines and regulations.

### Data collectives

For the reproducibility of our study, publicly available datasets are paramount. We therefore scanned the open-access journal “PLOS One” using the search key “radiomics” for papers that share their data publicly. Ten such studies have been identified and the data has been included into this study (Table 1). For reproducibility, all datasets were placed in a public repository (https://github.com/aydindemircioglu/radCV). All datasets were high-dimensional with two exceptions: Carvalho2018, which is the only low-dimensional dataset, and Song2020, which is almost low-dimensional. Here, we call a dataset high-dimensional if it has fewer samples than features, and low-dimensional otherwise.

**Table 1:** Overview of the datasets

Dataset	*N*	*d*	Dimensionality (#Samples/#Features)	Outcome balance (%)	Modality	Tumor type	DOI
Carvalho2018 [30]	262	117	2.22	59	FDG-PET	NSCLC	10.1371/journal.pone.0192859
Hosny2018A (HarvardRT) [31]	293	1004	0.29	54	CT	NSCLC	10.1371/journal.pmed.1002711
Hosny2018B (Maastro) [31]	211	1004	0.21	28	CT	NSCLC	10.1371/journal.pmed.1002711
Hosny2018C (Moffitt) [31]	183	1004	0.18	73	CT	NSCLC	10.1371/journal.pmed.1002711
Ramella2018 [32]	91	242	0.37	55	CT	NSCLC	10.1371/journal.pone.0207455
Toivonen2019 [33]	100	7105	0.01	60	MRI	Prostate Cancer	10.1371/journal.pone.0217702
Keek2020 [34]	273	1322	0.21	40	CT	HNSCC	10.1371/journal.pone.0232639
Li2020 [35]	51	396	0.13	63	MRI	Glioma	10.1371/journal.pone.0227703
Park2020 [36]	768	940	0.82	24	US	Thyroid Cancer	10.1371/journal.pone.0227315
Song2020 [37]	260	264	0.98	49	MR	Prostate Cancer	10.1371/journal.pone.0237587

Overview of all radiomics datasets used. Only publicly available datasets were included to allow for easy reproducibility. *N* denotes the sample size, while *d* denotes the number of features (corresponding to the dimension of the data). The outcome balance measures the number of events in the outcome. DOI denotes the identifier of the publication corresponding to the dataset

For each dataset, all available data, even if it was previously split into training and validation sets, was merged. This was performed to minimize any effect of non-identically distributed data on the prediction, which would potentially introduce a different bias. In the same spirit, all clinical features were removed, as the focus was only on the highly redundant and correlated radiomics features. More details can be found in Additional file 1.

### Cross-validation

Since some imbalance in the outcome was seen in a few datasets, stratified tenfold cross-validation was employed, i.e. while splitting of each dataset into 10 evenly sized folds it was made sure that the outcome balance in each fold was similar to the balance of the whole dataset. Cross-validation scores were computed by micro-averaging, i.e. first predictions from the 10 folds were pooled and then relevant metrics like AUC-ROC were computed on the pooled data.

### Preprocessing

An important step is the preprocessing of data whose main task is to harmonize the data. To avoid positive bias, especially in the presence of outliers, preprocessing must also take place inside the cross-validation. Despite this, in this study preprocessing was applied before the cross-validation to the whole dataset. This was done because application of preprocessing steps inside the cross-validation might interfere and occlude the bias arising from the incorrect application of feature-selection before the cross-validation. Since this effect was the main focus, preprocessing was performed upfront on the whole dataset.

Two preprocessing steps were applied: Imputation and normalization. The imputation was necessary as a few datasets had missing values. Such missing values can occur when computing large numbers of radiomics features because of numerical problems. The number of missing values was well below 1% for each feature and dataset. Imputation was performed by using column-wise means. Normalization using z-scores was applied afterwards.

### Feature selection

The goal of a feature selection is to remove redundant and irrelevant features. Redundant features are those which can equally well be expressed by other features, while irrelevant features are those which do not contribute to the performance of the model. While redundancy only depends on the data itself and not on the outcome, relevancy is only defined in relation to the outcome. Since the outcome is used in a very central way, removing irrelevant features can lead to a high positive bias if applied incorrectly.

The following 7 feature selection methods were used during modeling: LASSO, t-Score, f-Score, MRMRe (Minimum Redundancy, Maximum Relevance ensemble), ReliefF, MIM (Mutual Information Maximization) and SVM-RFE (Support Vector Machines-Recursive Feature Elimination). All these methods are filtering methods, i.e., they were applied before classification. Each method yielded a scoring on the features, based on which the best features were then selected. The number of selected features was chosen among 1, 2, 4, 8, 16 and 32. More information on the feature selection methods can be found in Additional file 1.

### Classifiers

Since the classifier is the ‘heart’ of radiomics, its choice is very important. Six classifiers which can be considered state-of-the-art were used: Logistic regression, random forests (RF), support vector machines with radial basis function kernel (RBF-SVM), neural networks (NN), XGBoost and Naive Bayes with Gaussian likelihood function. Each of these classifiers, with the exception of Naive Bayes, has its own hyperparameters, which were chosen from a predefined grid. In addition, a simple, constant classifier was employed that always predicted the majority class. This acts as a baseline which does not depend on the feature selection method. More information on the classifiers can be found in Additional file 1.

### Training schemes

**Fig. 1:**
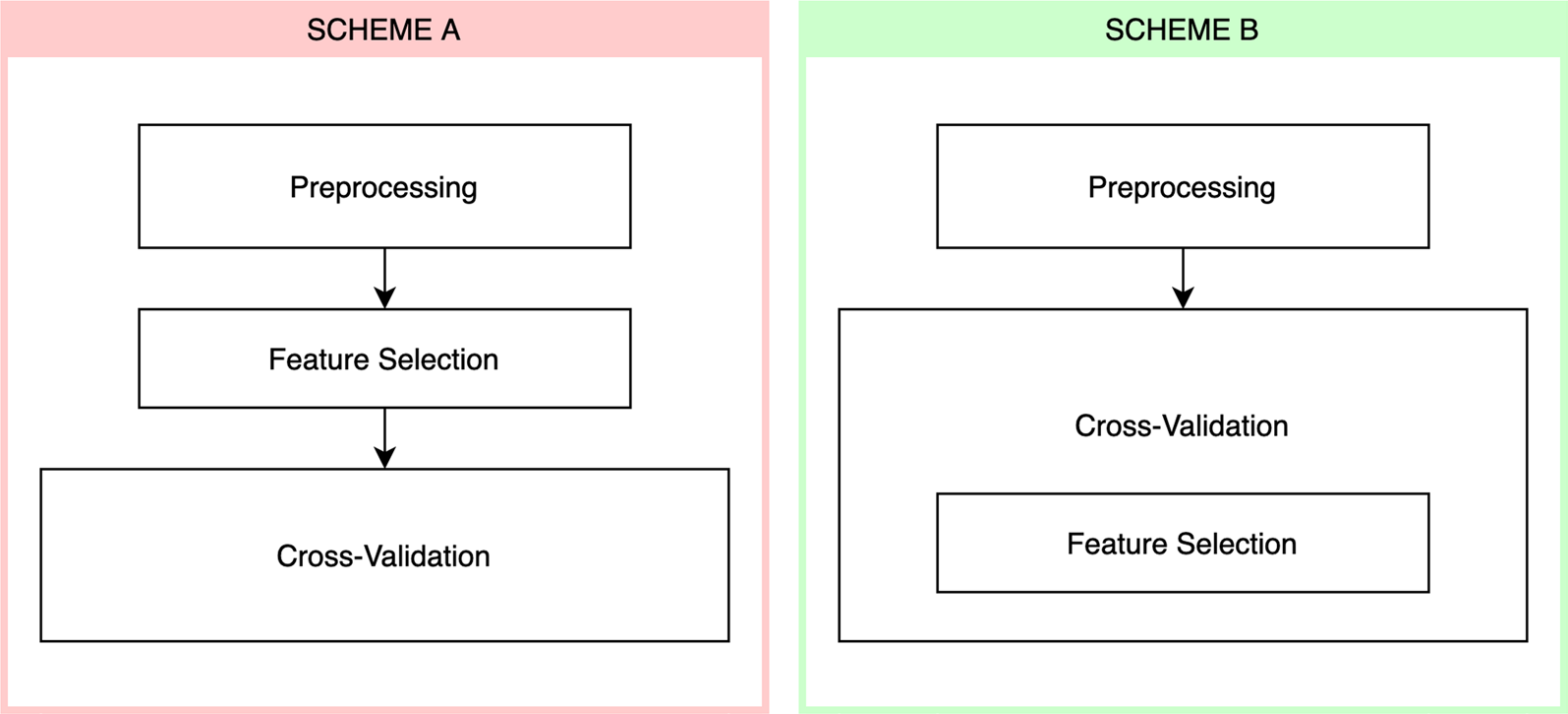
Illustration of the two training schemes used. In scheme A the feature selection is performed on the whole dataset before the cross-validation, while in scheme B the feature selection is part of the cross-validation, i.e. is applied only to each training fold. Note that preprocessing actually must be part of the cross-validation, but as it would interfere with the bias coming from applying the feature selection incorrectly, it was applied to all data

Two different training schemes were employed: First, the feature selection was applied to the whole dataset before the cross-validation (Fig. 1, Scheme A). This is incorrect, since the validation fold of the cross-validation was already used for feature selection. Second, the feature selection was correctly applied during the cross-validation separately in each fold (Fig. 1, Scheme B). Because of this, none of the validation folds in the cross-validation were used for feature selection and estimation is therefore not biased by data leakage.

### Evaluation

The performance of each model was measured primarily by AUC-ROC, since in many radiomics analyses AUC-ROC is chosen as the primary metric. More concretely, the model with the highest AUC-ROC using scheme A was selected and compared to the model with the highest AUC-ROC using scheme B. The difference in performance between these two models can be regarded as the bias of incorrectly applying feature selection before cross-validation.

Since higher dimensionality of a dataset, given by the ratio of the number of features to the number of samples, could influence the observed bias, the difference in AUC-ROC between the two schemes was plotted against the dimensionality. Linear regression was then applied to test if a significant relationship exists between both.

Finally, to understand how far different feature selection methods and classifiers are more prone to bias, we computed the difference in AUC-ROC between the best models for a given feature selection and classifier combination using scheme A and scheme B. This mimics studies that consider only a single feature selection method and classifier without an extensive search.

In addition to AUC-ROC, the AUC-F1, the area under the precision-recall-curve and the accuracy were also evaluated, but were only considered to be secondary. Other derived metrics, namely sensitivity, specificity, precision and recall and accuracy, were also computed and can be found in Additional file 2.

### Statistics

All descriptive statistics were reported as mean ± standard deviation. To compare the AUC-ROC, AUC-F1 and accuracy values of two models, bootstrap tests with 2000 repeats were employed. Statistical significance was chosen to be below a p-value of 0.05. Correlation coefficients were computed using Pearson's method. All analyses were conducted with Python 3.6.9 and the scikit learn 0.24.2 package.

## Results

**Fig. 2:**
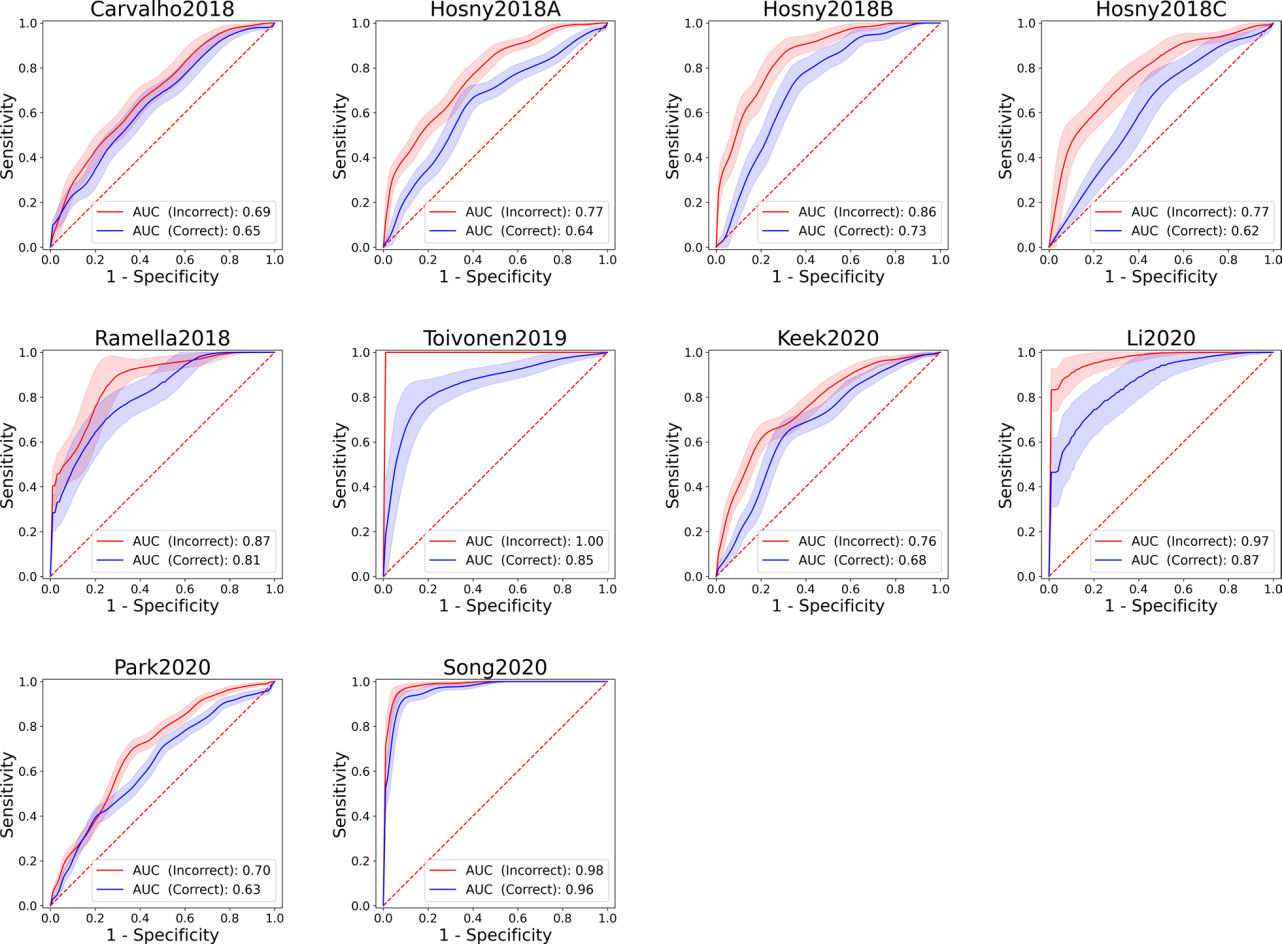
ROC curves for all datasets. The red and blue curves correspond to application of the feature selection before (Scheme A) and within (Scheme B) the cross-validation

Altogether, over 50,000 models have been fitted to the 10 datasets. Considering the best model in terms of AUC-ROC for each dataset, applying the feature selection incorrectly before the cross-validation led always to a positive bias when compared to the correct application of feature selection inside the cross-validation (Table 2). For AUC-ROC, the largest difference was seen for the Hosny2018C dataset (ΔAUC-ROC = 0.149) and the smallest one for Song2020 (ΔAUC-ROC = 0.02) (Fig. 2). Based on a bootstrap test for comparing AUC-ROCs of two ROC curves, the difference was highly significant for all datasets (*p* < 0.005) except for Li2020 (*p* = 0.018), and not significant for Carvalho2018 (*p* = 0.33) and Ramella2018 (*p* = 0.147).

**Table 2:** Results of the experiment

	AUC-ROC	ΔAUC-ROC	*P*	AUC-F1	ΔAUC-F1	*P*	Accuracy	ΔAccuracy	*P*
Carvalho2018 (Scheme A)	0.687	0.041	0.33	0.733	0.011	0.791	0.634	− 0.004	0.913
Carvalho2018 (Scheme B)	0.646			0.722			0.637		
Hosny2018A (Scheme A)	0.765	0.13	**< 0.001**	0.781	0.135	**0.001**	0.689	0.075	**0.035**
Hosny2018A (Scheme B)	0.636			0.647			0.614		
Hosny2018B (Scheme A)	0.855	0.13	**< 0.001**	0.716	0.293	**< 0.001**	0.791	0.09	**0.001**
Hosny2018B (Scheme B)	0.725			0.422			0.701		
Hosny2018C (Scheme A)	0.77	0.149	**0.005**	0.87	0.043	0.212	0.792	0.093	**0.019**
Hosny2018C (Scheme B)	0.621			0.827			0.699		
Ramella2018 (Scheme A)	0.872	0.061	0.147	0.893	0.051	0.21	0.846	0.11	**0.024**
Ramella2018 (Scheme B)	0.811			0.842			0.736		
Toivonen2019 (Scheme A)	1	0.146	**0.002**	1	0.038	**0.015**	0.98	0.17	**< 0.001**
Toivonen2019 (Scheme B)	0.854			0.962			0.81		
Keek2020 (Scheme A)	0.765	0.086	**0.005**	0.714	0.14	**0.001**	0.725	0.07	**0.018**
Keek2020 (Scheme B)	0.678			0.575			0.656		
Li2020 (Scheme A)	0.972	0.107	**0.018**	0.984	0.067	0.057	0.922	0.157	**0.006**
Li2020 (Scheme B)	0.865			0.917			0.765		
Park2020 (Scheme A)	0.698	0.067	**0.006**	0.394	0.061	**0.036**	0.763	0.005	0.602
Park2020 (Scheme B)	0.631			0.333			0.758		
Song2020 (Scheme A)	0.985	0.02	**0.002**	0.984	0.022	**0.007**	0.942	0.012	0.334
Song2020 (Scheme B)	0.965			0.962			0.931		

AUC-ROC, AUC-F1 and accuracy of the correct and incorrect models for each dataset as well as their differences and significance. The *p*-values were computed using a bootstrap test with the null hypothesis that the difference is zero. Significant *p*-values are marked in bold

Similarly, in the AUC-F1 as well as the accuracy a positive bias up to 0.293 and 0.17 respectively, could be seen with the only exception of Carvalho2018, which is the only low-dimensional dataset, where a very minor bias in AUC-F1 was seen (ΔAUC-F1 = 0.011) and even a slight loss in accuracy (ΔAccuracy =  − 0.004).

**Fig. 3:**
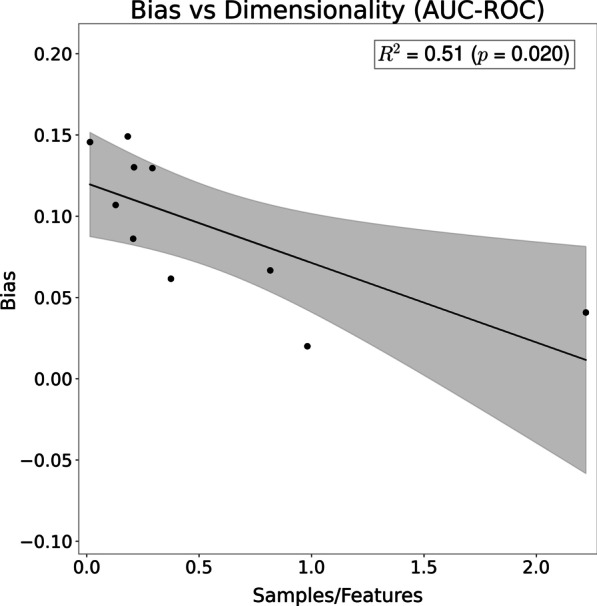
Scatter plot relating the number of samples per feature against the observed bias in AUC-ROC, when feature selection is applied incorrectly, for each dataset

Plotting the samples per feature number against the observed bias showed a significant negative tendency for AUC-ROC (Pearson correlation *R* =  − 0.72; *p* = 0.02), indicating that with fewer samples per feature the likelihood of bias increases (Fig. 3). Similar tendencies could be seen for the F1-score (*R* =  − 0.64, *p* = 0.045) and accuracy (*R* =  − 0.77, *p* = 0.008).

**Fig. 4:**
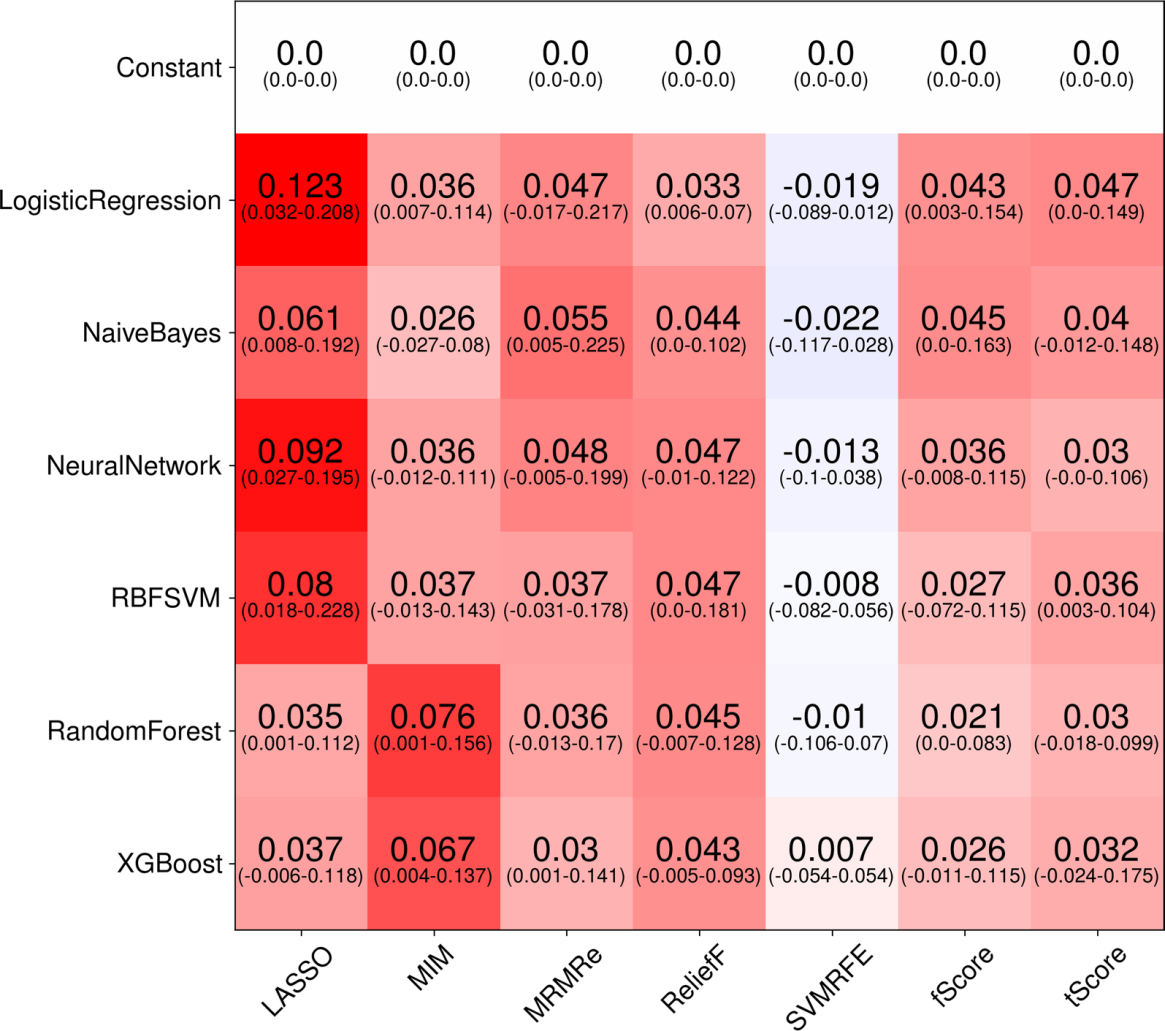
Mean bias in AUC-ROC for each combination of feature selection and classification method over all datasets. To obtain these, for each of the 10 datasets, the largest difference in AUC-ROC for a given combination between correct and incorrect application of CV is computed, resulting in 10 differences. The mean of these differences is denoted, with the corresponding ranges below in parentheses. Since the displayed mean is an average of all datasets, a conclusion about the bias for a single dataset is not possible. For example, using SVM-RFE with a random forest shows almost no bias in mean (− 0.01), but the difference for a single dataset can be as high as 0.07 in AUC-ROC

To understand how far the bias can be traced back to the feature selection method and the classifier, for each dataset the best AUC-ROC of each combination was considered. Then, the difference to the AUC-ROC of the best model with the same combination, but with incorrectly applied feature selection was measured. The mean of these differences over all dataset was then computed (Fig. 4). The results show that especially LASSO, especially with Logistic regression, RBF-SVM and Neural Networks as classifiers, as well as MIM tend to show high bias, if feature selection is incorrectly applied. On the other hand, it would seem that SVM-RFE is less biased. But even here, e.g. when the SVM-RFE is combined with random forests, on Toivonen2019 a bias of 0.07 in AUC-ROC can be observed.

The average bias in F1-score was also very high for the LASSO and MIM, and equally lower for the SVM-RFE. Accuracy showed less overall bias, again LASSO shows larger bias than other feature selection methods.

## Discussion

Obtaining reliable models and predictions in radiomics is notoriously difficult because of the high dimensionality of the datasets involved. Accordingly, several guidelines were presented [12, 13] and a radiomics score was introduced to safeguard against spurious results and to define best practices [14]. Despite this, it is not evident if all radiomics studies follow best practices.

We have studied how far an incorrectly applied feature selection on the whole dataset before cross-validation leads to a bias because of data leakage. Our results clearly showed that a large positive bias can result from this that can be as high as 0.15 in AUC-ROC, which is the primary metric in many radiomics studies. This underlines the fact that feature selection applied to the whole dataset induces a large bias that must be avoided at any cost, if results should be trusted.

While all models showed a positive bias in AUC-ROC when feature selection was applied incorrectly, the datasets Toivonen2019 and Hosny2018 stand out. On Toivonen2019, the incorrect model yielded an AUC-ROC of 1.0, in stark difference to the analysis by Toivonen et al. which yielded an AUC-ROC of 0.88, comparable to the AUC-ROC of 0.86 we obtained [33]. Similarly, on Hosny2018A and Hosny2018C, predictions were not much different from a random guess (AUC-ROC of 0.62 and 0.64), but became apparently quite usable when using the incorrectly applied feature selection (AUC-ROC of 0.77), in contrast to the study by Hosny et al., since they reported an AUC-ROC of 0.66 [31]. Similar trends could be seen for AUC-F1, although the high bias of 0.293 stemmed from a particularly low AUC-F1 for Scheme B (0.422), which was worse than a random guess. Such performance can happen as we did not select the best model for AUC-F1, but for AUC-ROC. Accuracy also showed a positive bias up to 0.17, but was non-significant on three datasets.

From the experiment, it seemed that indeed datasets with higher dimensionality, i.e. more features per sample, were more prone to overfitting when feature selection was applied incorrectly. This was to be expected as feature selection is hard and even small data leakage can help to select better features, resulting in a positive bias.

Considering whether certain feature selection methods were more prone to bias than others, it seemed that on average LASSO and MIM showed more positive bias, while SVM-RFE behaved better in this regard. Still, even SVM-RFE showed a positive bias of 0.07 on Toivonen2019. This bias corresponds to an additional 20 patients (of 100) being incorrectly classified as having a prostate cancer with Gleason score > 3 + 3, instead of  3 + 3 (Biased: TN = 10, FN = 6, FP = 10, TP = 74 vs. unbiased: TN = 13, FN = 26, FP = 7, TP = 54). Thus, the seemingly small average bias cannot be used as a pretext to circumvent correct application of features selection.

From the plot, it appeared that the feature selection has a much larger impact than the choice of the classifier, which at first sight contradicts the results by Parmar etc. [38]. This arises from the fact that we considered biases, not overall performances. Because the only difference between scheme A and scheme B was whether the feature selection method was able to select better features because of data leakage, this result appears to be reasonable.

Feature selection has been considered for long [39–41] and the effect of applying it outside of the cross-validation has been studied previously for more general datasets, but not for radiomics datasets. Refaeilzadeh et al. consider pair-wise comparison of feature selection algorithms in the setup of cross-validation, and argue that especially for small datasets correct application of cross-validation is wasteful as not all data is used for feature selection, thus inducing another bias. They conducted an experiment on low-dimensional synthetic datasets and showed both methods have different biases, up to 7%, and in the end, they do not differ significantly. Our results do not contradict these findings, since our datasets are high-dimensional, where it is known that the bias can be larger. In the same spirit, Aldehim and Wang considered 10 real-world and 14 synthetic datasets using 4 different feature selection methods and 3 classifiers [42]. They concluded that for datasets with large samples there is “no noticeable difference”, but for smaller datasets a bias has occurred”. Our experiments confirm this finding, we demonstrated that for radiomics datasets the bias is actually even larger than they have observed, possibly not only because of smaller sample sizes, but also because of highly correlated features.

Analysis of highly correlated features is difficult and can lead to spurious results: Using a cohort of patients with head and neck cancer, Ger et al. demonstrated that tumor volume alone obtained a higher AUC-ROC than a model based on radiomics features, and that the combination of both surprisingly decreased the performance [43]. In the same spirit, Welch et al. showed that three out of four features of the seminal radiomics model presented by Aerts et al. [1] highly correlated with tumor volume and that tumor volume alone yields the same performance, basically questioning whether radiomics beyond tumor volume has any benefit at all in this case [44].

Our focus was to show that an incorrect feature selection indeed leads to a large positive bias when compared to the correct application of feature selection. However, some limitations apply to our study. Foremost, without explicit independent validation sets, the true extent of the bias cannot be determined. A reasonable solution would have been to either split these off from the datasets or to use validation sets where given. Since such validation sets were not always available, and because of the low sample sizes of the datasets, we were unable to do this.

Regarding the experiments, several choices had to be made. We tried to use more common feature selection and classifiers but only tuned a few of the multitude of hyper-parameters because of computational restrictions. It can be expected that better tuning will lead to even higher bias.

Technically, the normalization and also the imputation of missing values must also be part of the cross-validation. In this study, we forfeit this and applied the normalization and imputation as preprocessing steps to avoid another source of bias, since both do not use the outcome, and thus the influence of them should be rather small when compared to the feature selection. For the same reason, other techniques which are often used in radiomics studies, like outlier removal, or synthetically generating additional training samples to overcome imbalanced problems were not applied. Therefore, our results can be understood as a lower estimate to the bias.

There are still open questions, for example, we used tenfold cross-validation, and it is not clear how a different validation scheme like a fivefold CV or leave-one-out CV will impact the observed bias. However, such studies would not change the fact that incorrect application of feature selection and cross-validation is self-evidently wrong, regardless of the bias that could or could not occur. They can only increase the awareness of this problem since this kind of misapplication seems still to be present in recent studies.

## Conclusion

We have shown that incorrectly applying feature selection before cross-validation to high-dimensional radiomics data can lead to positive bias because of data leakage.

## Supplementary Information

**Additional file 1.** Details on the datasets and experiments performed.

**Additional file 2.** Results of all experiments.

## Data Availability

All datasets generated and/or analyzed during the current study are publicly available (https://github.com/aydindemircioglu/radCV).

## Abbreviations

AUC: Area under the curve
AUC-F1: Area under the precision-recall curve
AUC-ROC: Area under the curve of receiver-operating characteristics
CT: Computed tomography
CV: Cross-validation
FDG-PET: Fluorodeoxyglucose-positron emission tomography
MIM: Mutual information maximization
MRMRe: Minimum redundancy, maximum relevance ensemble
NN: Neural network
RBF-SVM: Support vector machine with radial basis function kernel (RBF-SVM)
RF: Random forests
SVM: Support vector machine
US: Ultrasound
