# Supplementary material for: Measuring the bias of incorrect application of feature selection when using cross-validation in radiomics
Source: Insights Imaging. 2021 Nov 24;12:172. doi: 10.1186/s13244-021-01115-1 (PMC8613324; doi:10.1186/s13244-021-01115-1)
Supplement: Supplementary file 1 — Additional file 1. Details on the datasets and experiments performed. [file 13244_2021_1115_MOESM1_ESM.docx]

**Supplemental 1**

***Datasets***

The following preprocessing steps were applied to the datasets:

**Carvalho2018**: The Radiomics.PET.features.csv file was used. Since the target is survival, it was converted to a binary endpoint as follows: First, patients who were censored and had less than 2 years follow-up time, were removed. Then, the two-years survival was taken as a binary target.

**Hosny2018A** (HarvardRT)/**Hosny2018B** (Maastro)/**Hosny2018C** (Moffitt): The HarvardRT.csv/ Maastro.csv/Moffitt.csv files were used with “surv2yr” as target. Logits were removed.

**Ramella2018**: The pone.0207455.s001.arff file was used with target “adaptive”. Other targets (e.g. T, N, mutation) were removed.

**Toivonen2019**: The lesion_radiomics.csv file was used with “gleason_group” as target.

**Keek2020**: For Keek2020, we only used the DESIGN data, as we found a mismatch between the clinical and radiological datasets for the DECIDE data, since the clinical data contained information of 143 patients, but only radiomics features for 116 patients could be found. For the DESIGN data, patients who were censored but had a follow-up time less than 3 years were removed, guaranteeing that every patient had at least 3 years of follow-up time. Then, the 3 years survival was taken as a binary target.

**Li2020**: The pone.0227703.s014.csv file was used.

**Park2020**: The pone.0227315.s003.xlsx file was used with the target “pathological lateral LNM 0=no, 1=yes”.

**Song2020**: The numeric_feature.csv file was used

Data is shared via github (https://github.com/aydindemircioglu/radiomicsCV).

***Feature selection***

The following parameters were chosen for the feature selection methods:

**LASSO**: The LASSO feature selection was applied by first fitting a LASSO regression to the data and then selecting the features with the highest absolute coefficients. The C parameter of the LASSO regression was chosen from [0.1, 1, 10, 100, 1000]. The ‘liblinear’ solver was used with a maximum of 500 iterations. The LogisticRegression from scikit-learn was used.

**t-Score**: t-Score is performed by computing the statistics of a t-test between each feature and the outcome and selecting those with the highest significance. The t-score function from the scikit-feature package was used (see folder “3rd”).

**f-Score**: f-Score is performed by computing the statistics of a f-test (ANOVA) between each feature and the outcome and selecting those with the highest significance. The f-score function from the scikit-feature package was used (see folder “3rd”).

**MIM:** No parameters were changed beyond the default parameters. The mutual_info_classif function from scikit-learn was used.

**ReliefF**: No parameters were changed beyond the default parameters. The relief function from the scikit-feature package was used (see folder “3rd”).

**MRMRe:** MRMRe was used with 5 solutions. Because the python package pymrmre did not produce a final solution, the 5 rankings were combined by a simple heuristics which weighted each selected feature by the rank in the solution. The mrmr_ensemble function of pymrmre was used.

**SVM-RFE**: For SVM-RFE a linear SVM with a fixed C value of 1, to avoid extensive computation times. In each round of SVM-RFE, 10% of all features with the lowest coefficients were removed. The RFECV function of scikit-learn was used, with all other parameters fixed.

***Classifiers***

The following parameters were used for the classifiers:

**Dummy Classifier**: No parameters. The DummyClassifier of scikit-learn was used.

**RBF-SVM**: The kernelized SVM was used with C between 2^-6 and 2^6. The gamma parameter was selected automatically (1/number of features). The SVC classifier of scikit-learn was used with rbf kernel.

**RandomForest**: Only the number of estimators were selected from [25, 100, 250]. Other parameters were kept fix. The RandomForestClassifier from scikit-learn was used.

**XGBoost**: The two parameters, the learning rate and the number of estimators, were tuned. The learning rate was chosen from [0.001, 0.1, 0.3, 0.9], while the number of estimators were taken from [25, 100, 250]. The XGBClassifier from the xgboost package was used.

**LogisticRegression**: The LogisticRegression of the scikit-learn package was used with LBFGS as solver.

**NeuralNetwork**: A network with two hidden layers were used, with sizes from [8, 16, 32] for each layer. The MLPClassifier from scikit-learn was used. Other parameters were kept fix, i.e., a ReLU activation function between the layers were used as well as the ADAM optimizer with a initial learning rate of 0.001.

**NaiveBayes**: The gaussian Naïve Bayes from the scikit-package was used, no parameters were changed.

***Experiments***

As the choice of the cross-validation folds is random and thus could have an effect on the bias, for both experiments the very same folds are chosen. For both experiments, we show the algorithms in pseudocode:

*# preprocess Data*

Impute NAs in data by mean, normalize data to z-scores.

*# fix folds of 10-fold CV*

Split data into 10 random folds with stratification

*# Scheme A: incorrectly apply first feature selection, then 10-fold CV*

Apply feature selection to X, obtain new dataset X_fs_

For each fold:

Train classifier on the training folds of X_fs_, evaluate on validation fold of X_fs_

Compute metrics over all validation folds by pooling the prediction scores

*# Scheme B: Apply feature selection in each fold*

For each fold:

Apply feature selection to training folds, to obtain X^train^_fs_

Select same features in the validation fold to obtain X^val^_fs_

Train classifier on X^train^_fs_, evaluate on validation fold X^val^_fs_

Compute metrics over all folds by pooling the prediction scores

This code can be found in the ‘*executeExperiment’* function in the *startExperiments.py* file. The comparison of the metrics is done for technical reasons in another script, *evaluate.py.*

In a few cases some errors occurred. For example, in the Ramella2018 dataset, it occurred a few times that the features selected were seemingly constant, so that the standard deviation was zero. The Naive Bayes classifier then raised a warning and produced NaN values. To avoid this, we replaced all NaN in the prediction by 0. Similarly, a division by zero warning occurred in the t-Score feature selection. We suppressed this error message.

Finally, the constant classifier did behave problematic, if the feature selection did not select any feature. This only happened for the LASSO. In this case, the constant classifier returned 0 instead of the probability of an event. To circumvent the problem, we just took the first features of the dataset, so that the constant classifier worked as expected.

All code was developed using Python 3.6.9 using *Ubuntu 20.04 LTS*—other platforms might need changes and might produce different results.

***Data availability***

All code, data and results are shared online (https://github.com/aydindemircioglu/radiomicsCV).
