# Supplementary material for: Measuring the bias of incorrect application of feature selection when using cross-validation in radiomics
Source: Insights Imaging. 2021 Nov 24;12:172. doi: 10.1186/s13244-021-01115-1 (PMC8613324; doi:10.1186/s13244-021-01115-1)
Supplement: Supplementary file 2 — Additional file 2. Results of all experiments. [file 13244_2021_1115_MOESM2_ESM.docx]

**Supplemental 2**

**Results Overview**

|  | **AUC** | **Sens** | **Spec** | **F1** | **Precision** | **Recall** | **Accuracy** |
| --- | --- | --- | --- | --- | --- | --- | --- |
| **Carvalho2018** | 0,041 | 0,032 | 0,019 | -0,055 | 0,049 | -0,221 | -0,004 |
| **Hosny2018A** | 0,13 | 0,044 | 0,045 | 0,049 | 0,079 | 0,013 | 0,075 |
| **Hosny2018B** | 0,13 | 0,033 | 0,126 | 0,18 | 0,225 | 0,15 | 0,09 |
| **Hosny2018C** | 0,149 | -0,008 | 0,16 | 0,067 | 0,04 | 0,098 | 0,093 |
| **Ramella2018** | 0,061 | 0,08 | 0,098 | 0,105 | 0,073 | 0,14 | 0,11 |
| **Toivonen2019** | 0,146 | 0,213 | 0,1 | 0,104 | 0,133 | 0,075 | 0,17 |
| **Keek2020** | 0,086 | -0,025 | 0,123 | 0,098 | 0,081 | 0,109 | 0,07 |
| **Li2020** | 0,107 | 0,188 | 0,105 | 0,117 | 0,173 | 0,062 | 0,157 |
| **Park2020** | 0,067 | -0,022 | 0,144 | 0,193 | 0,108 | 0,137 | 0,005 |
| **Song2020** | 0,02 | 0 | 0,03 | 0,011 | 0,029 | -0,008 | 0,012 |

**ROC Curves**

**
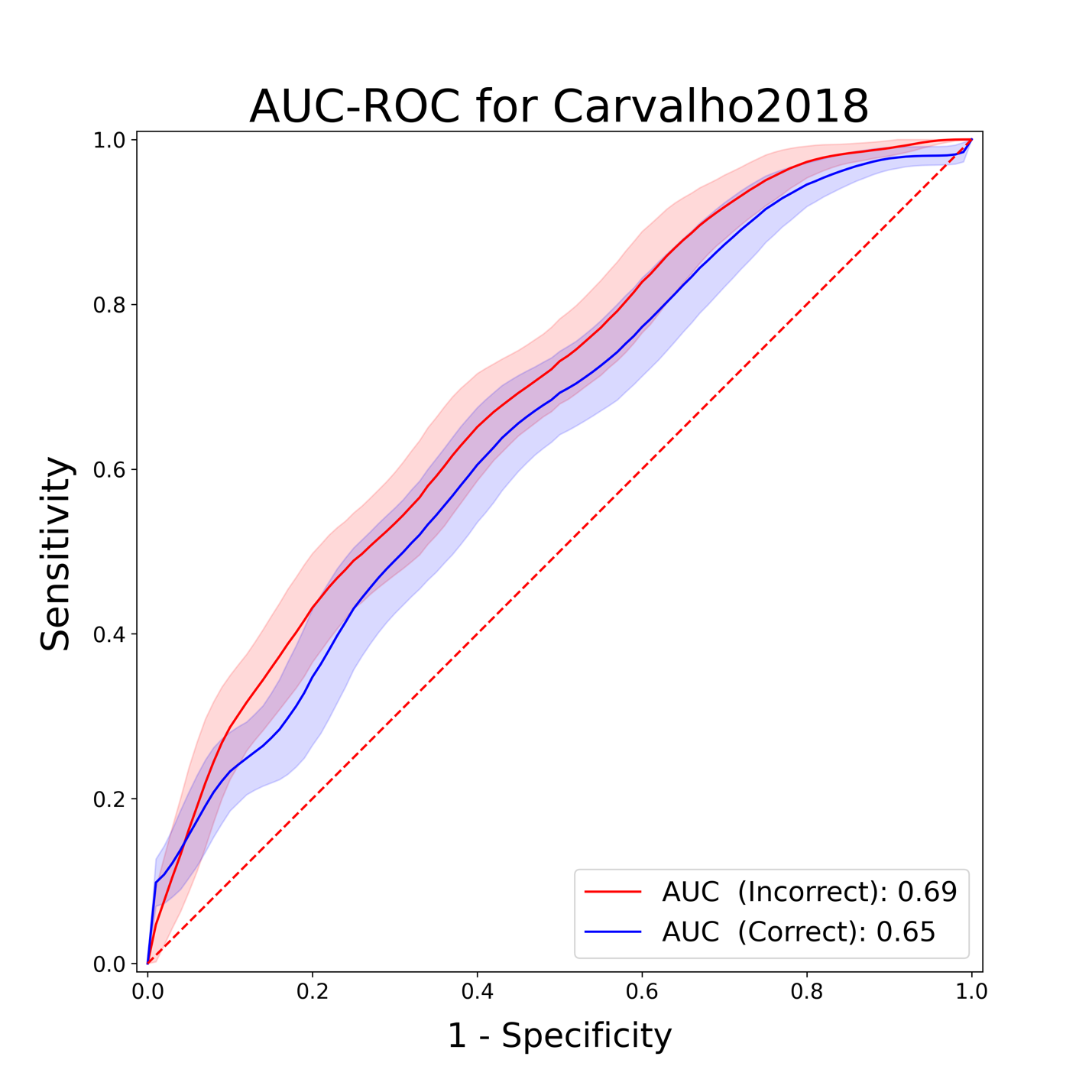

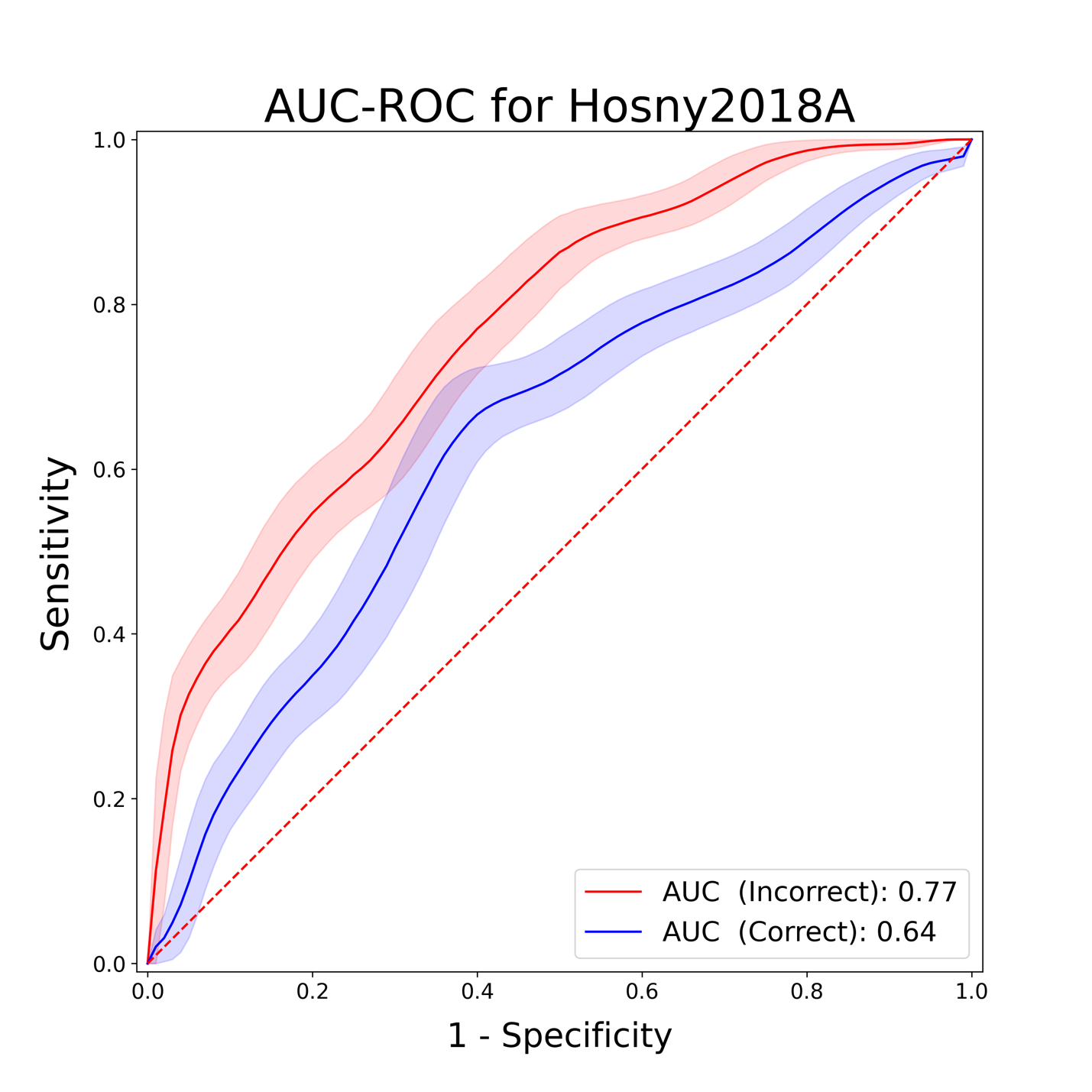

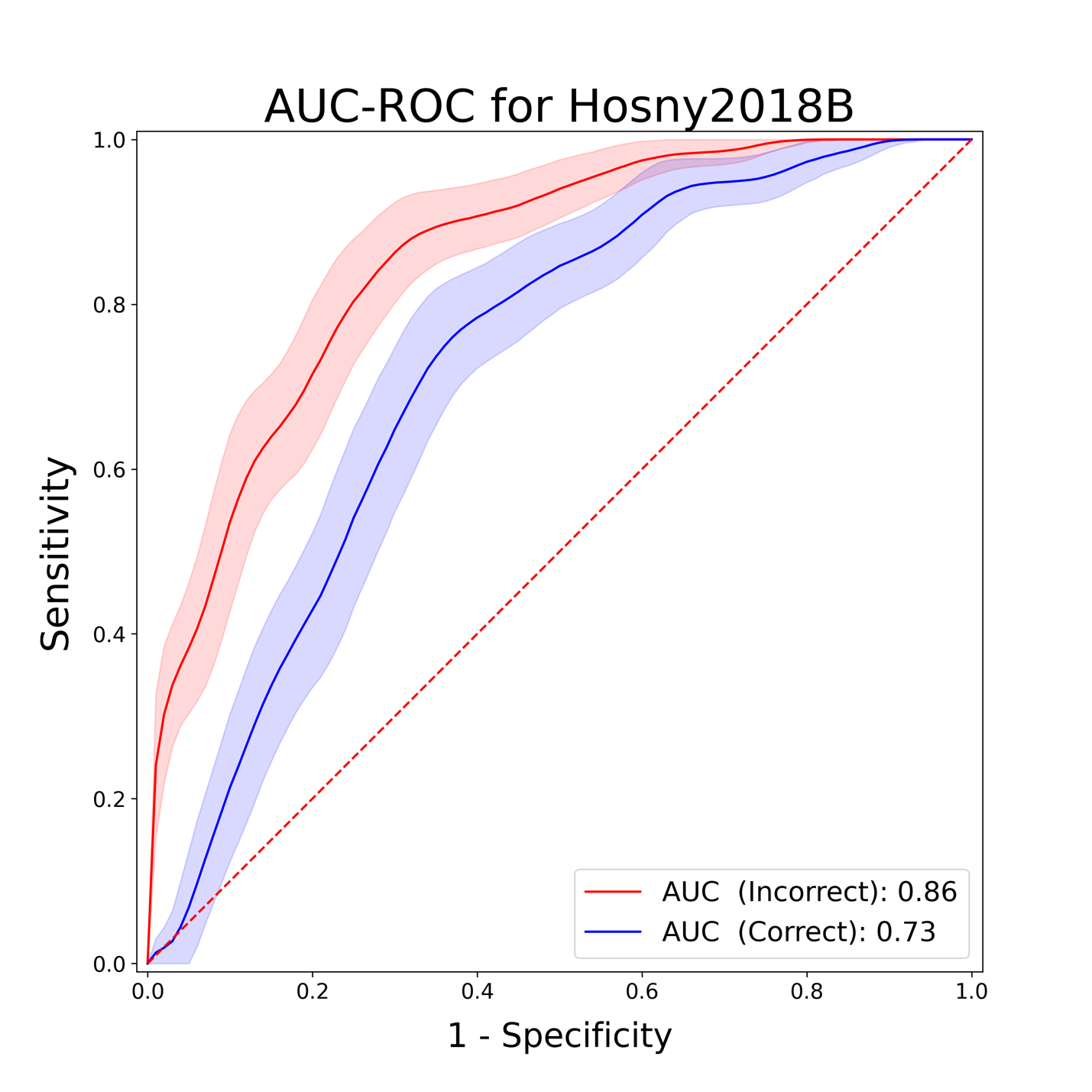

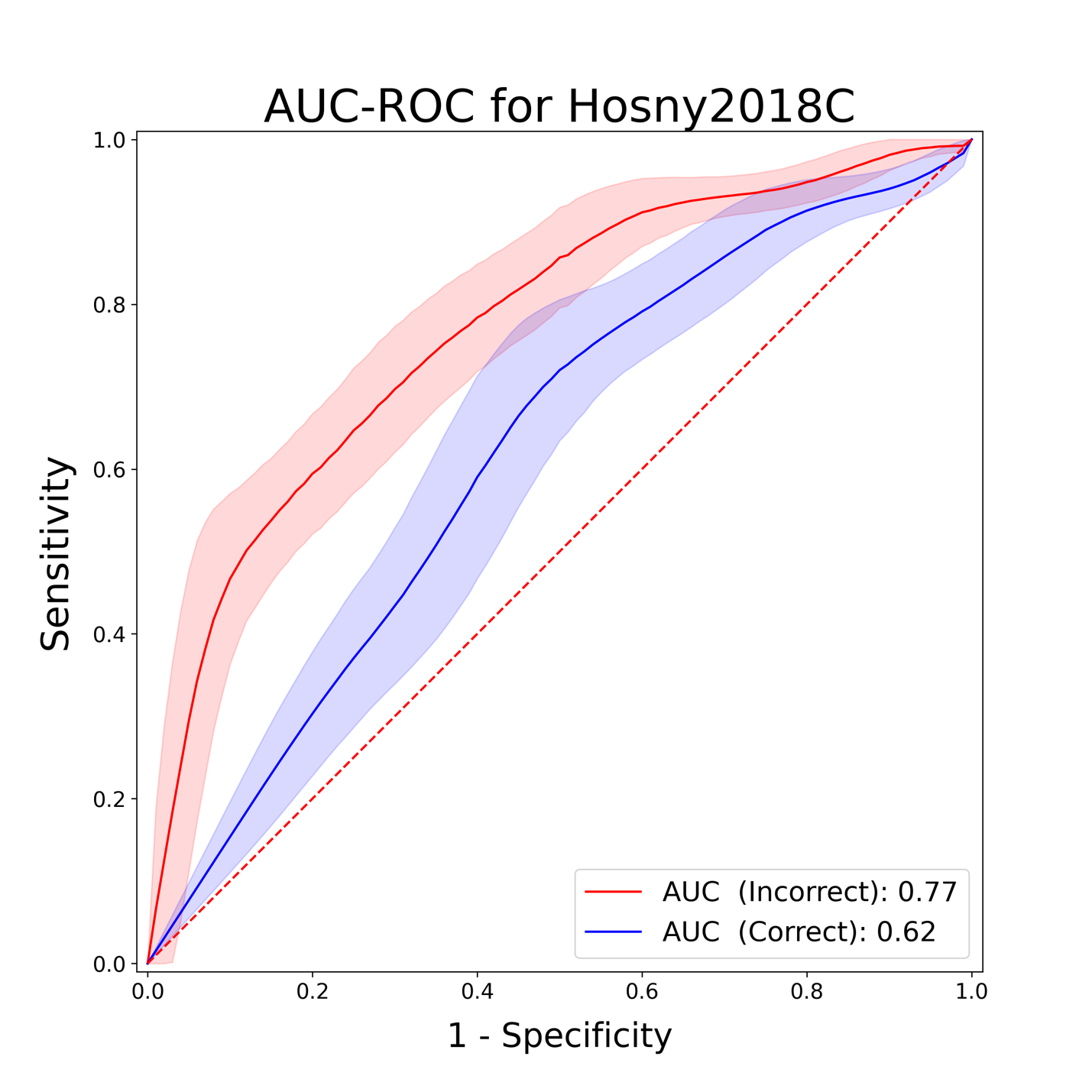

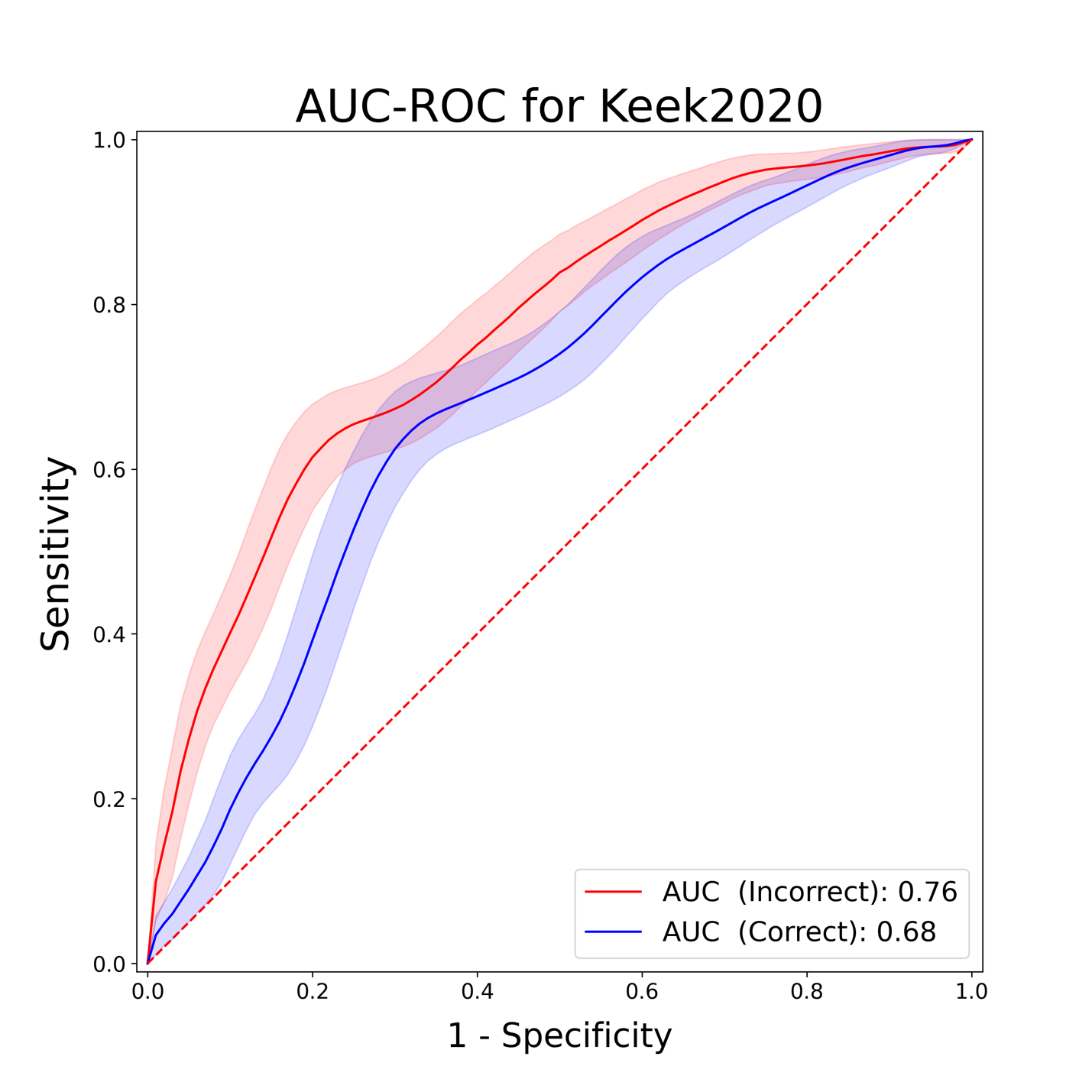

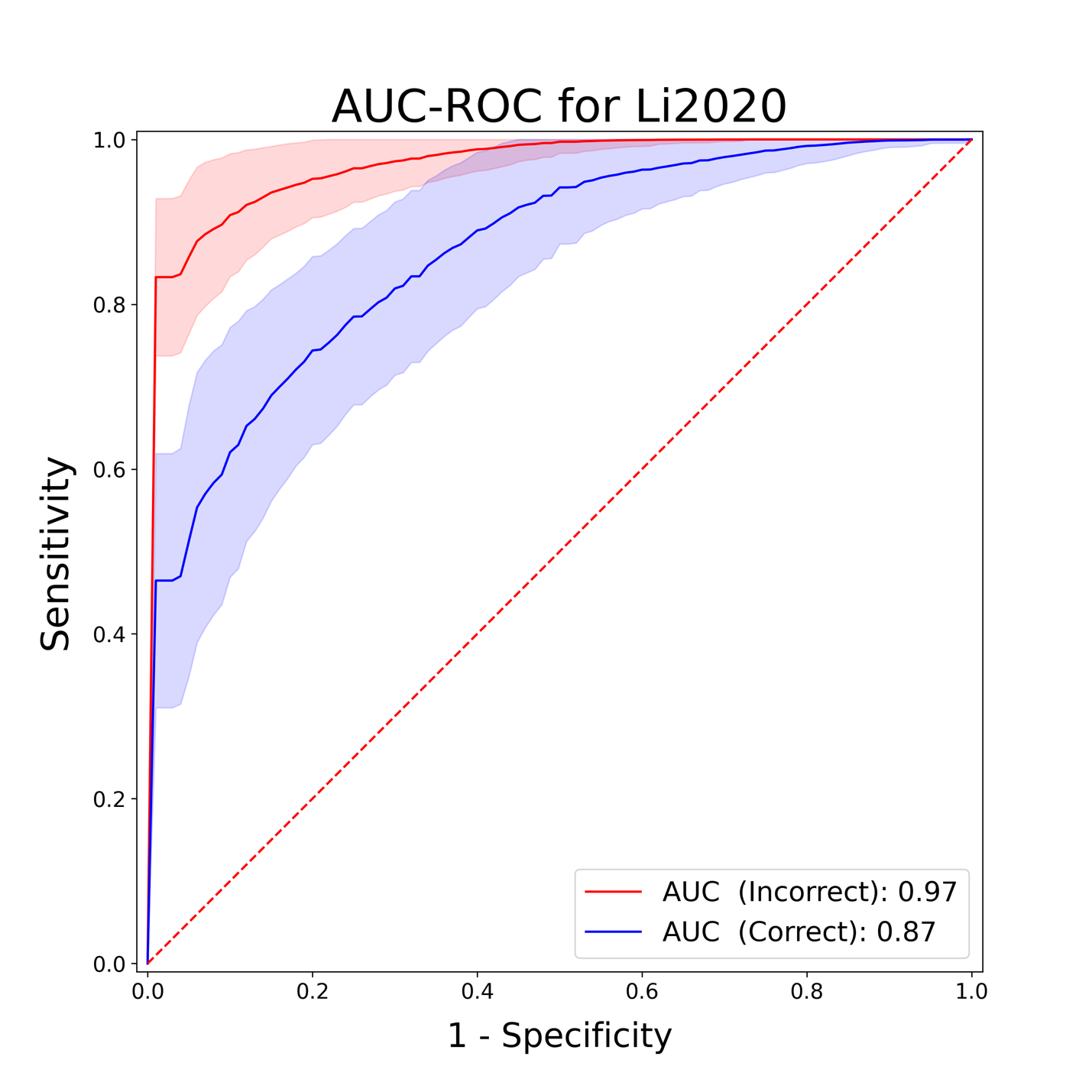

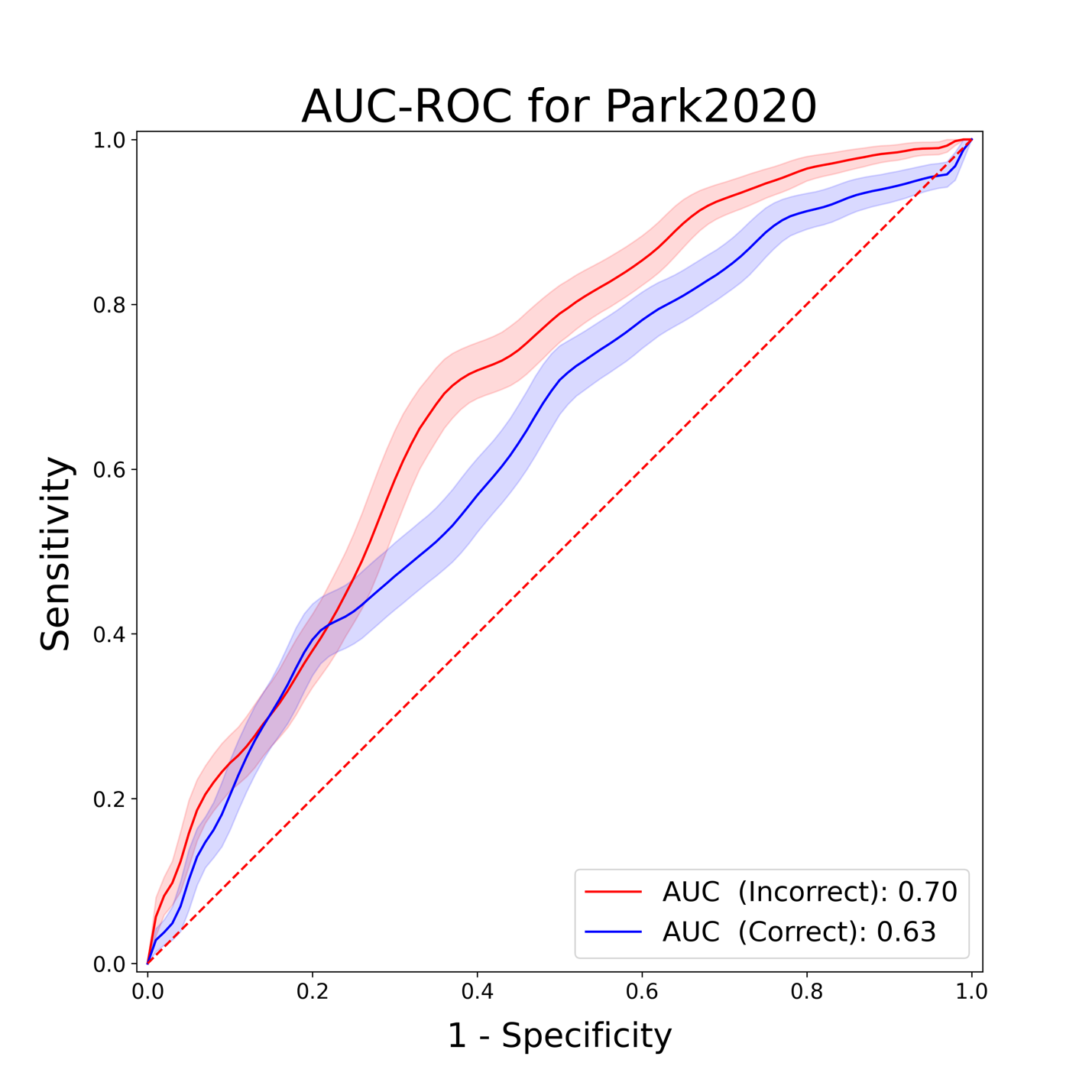

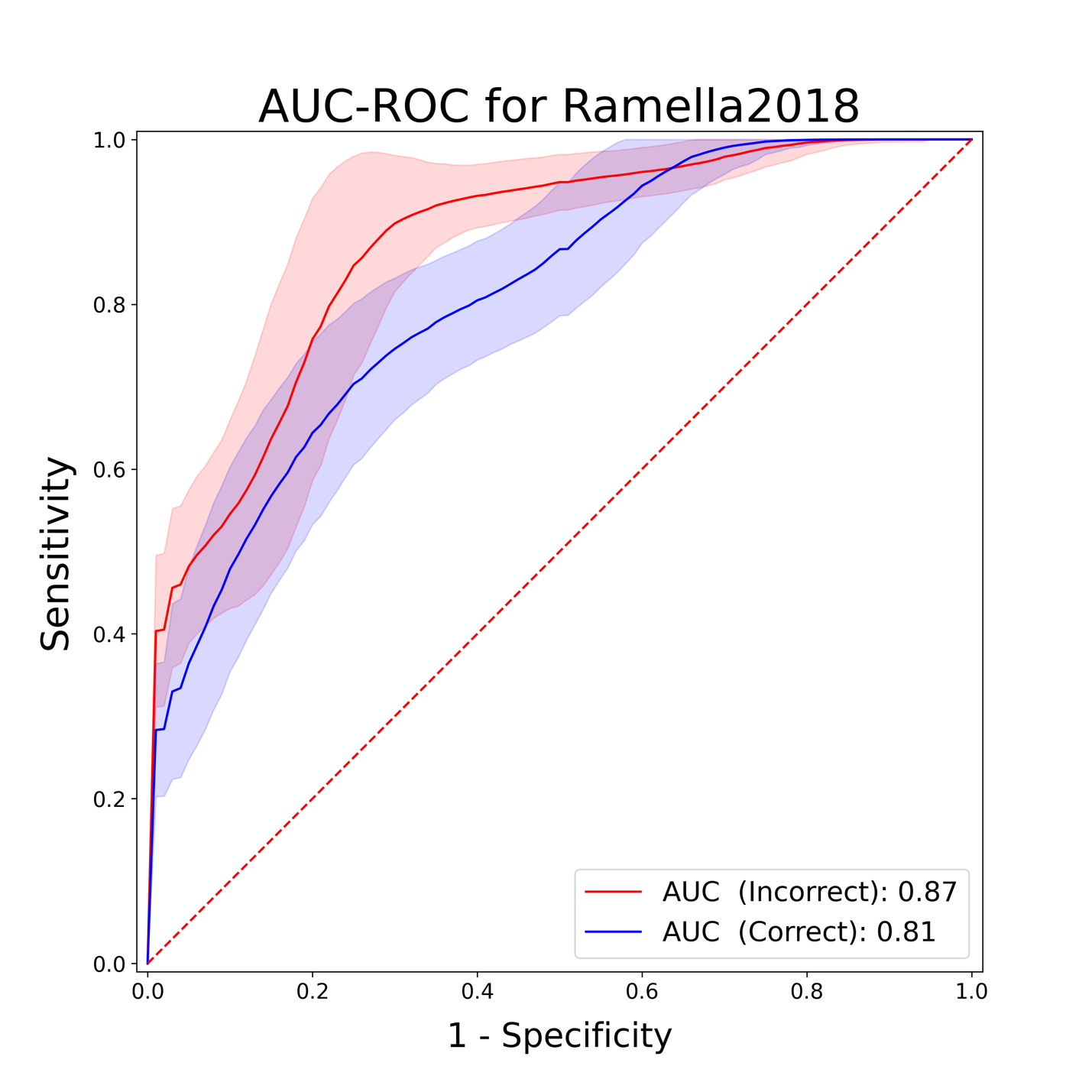

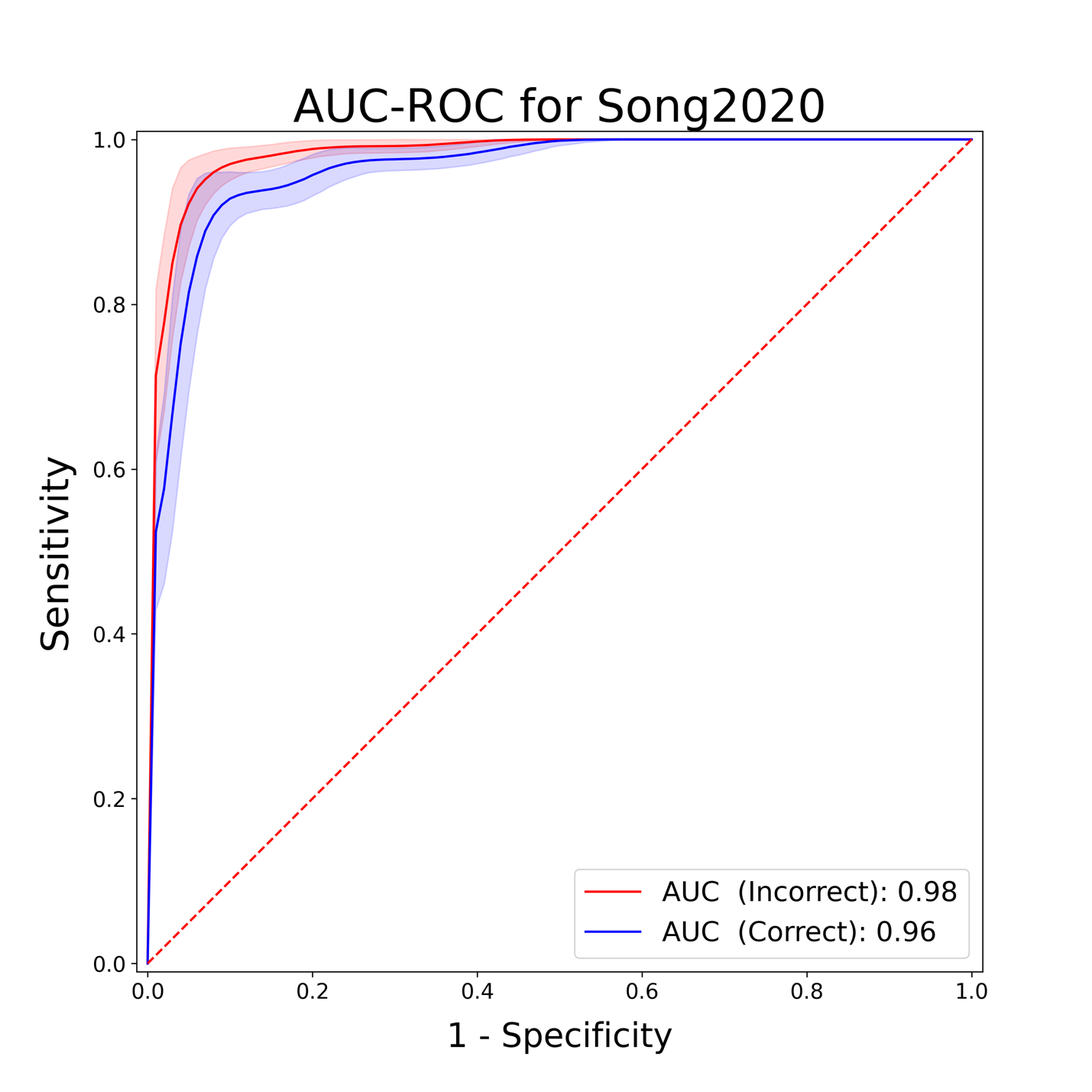

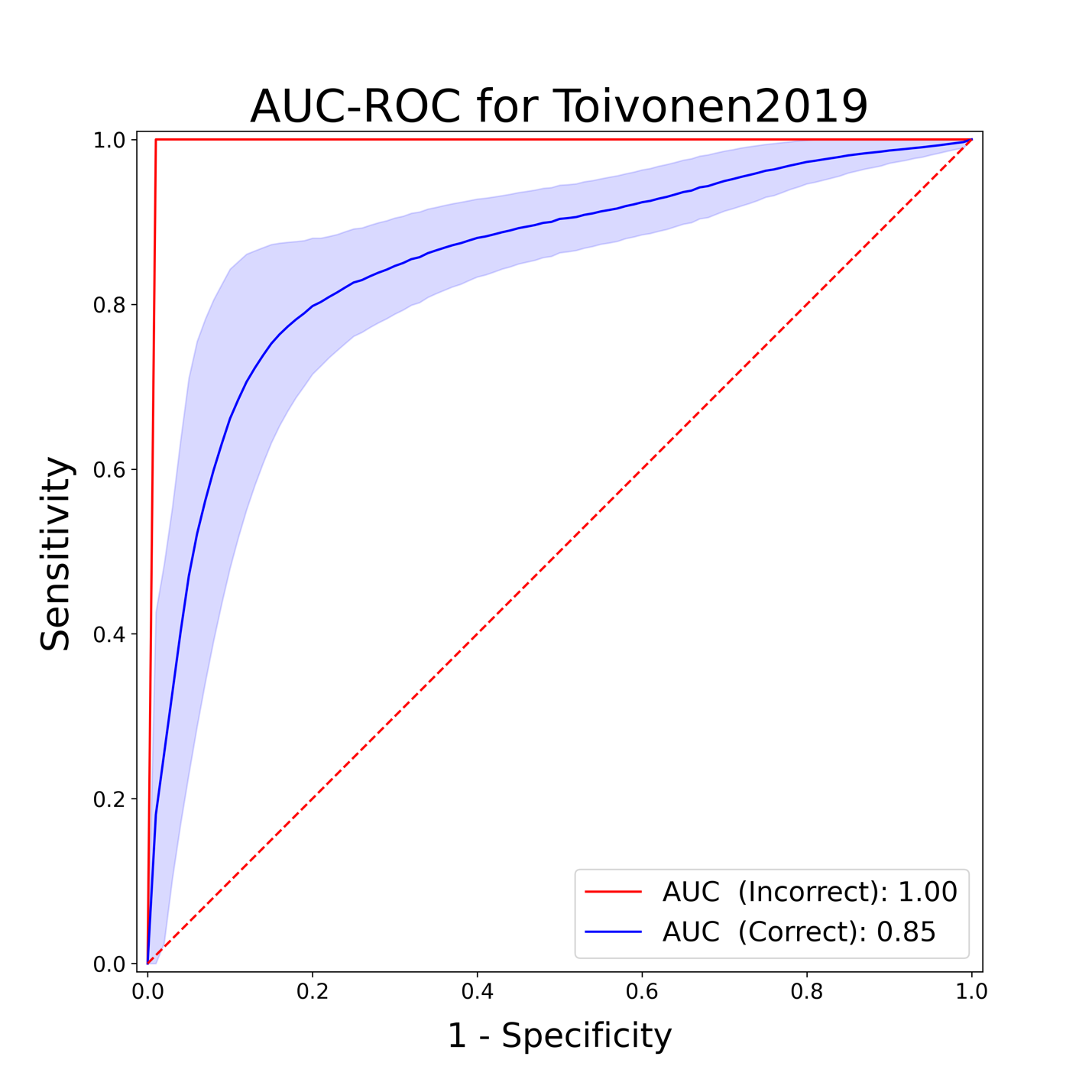

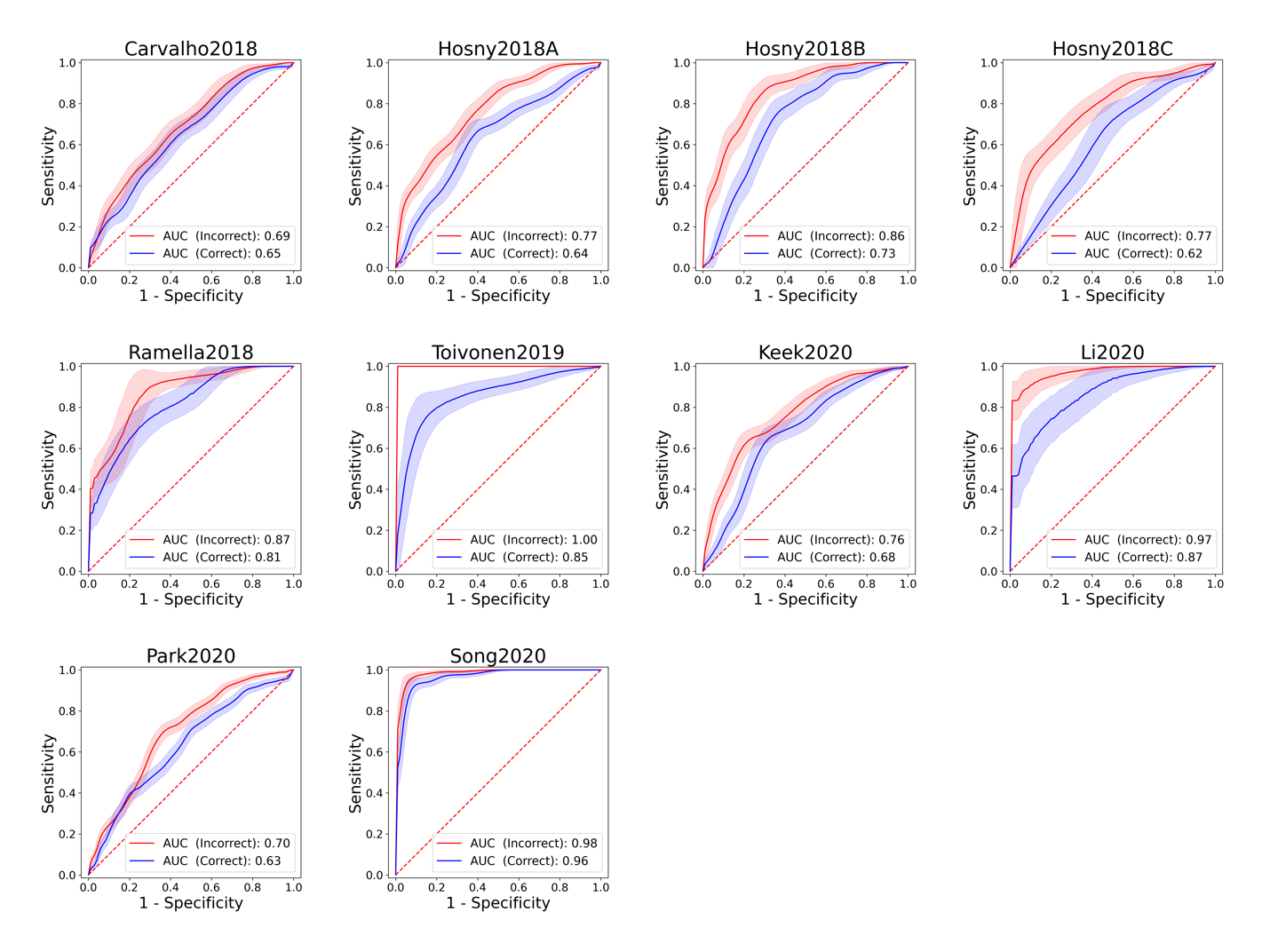
**

**PR Curves**

**
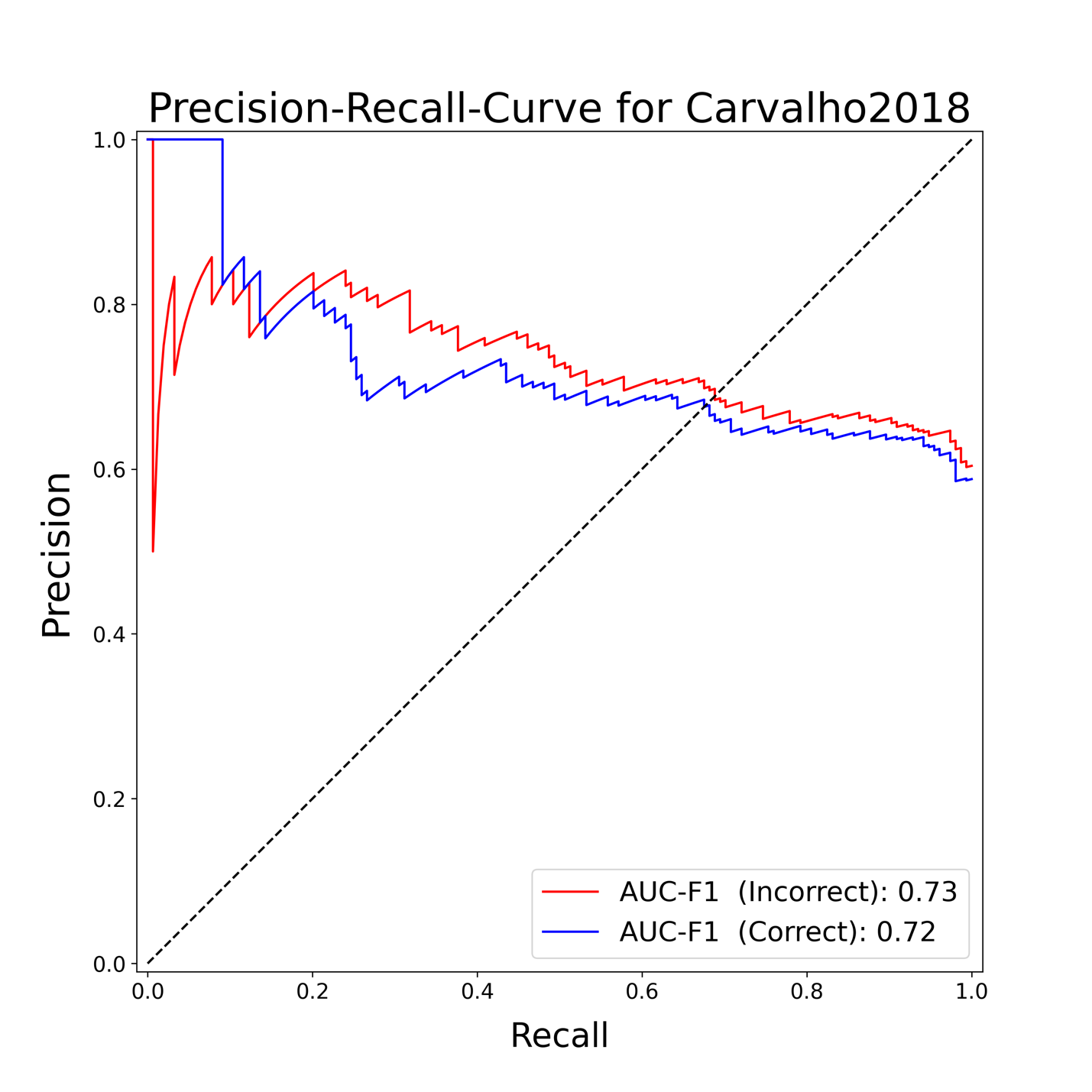

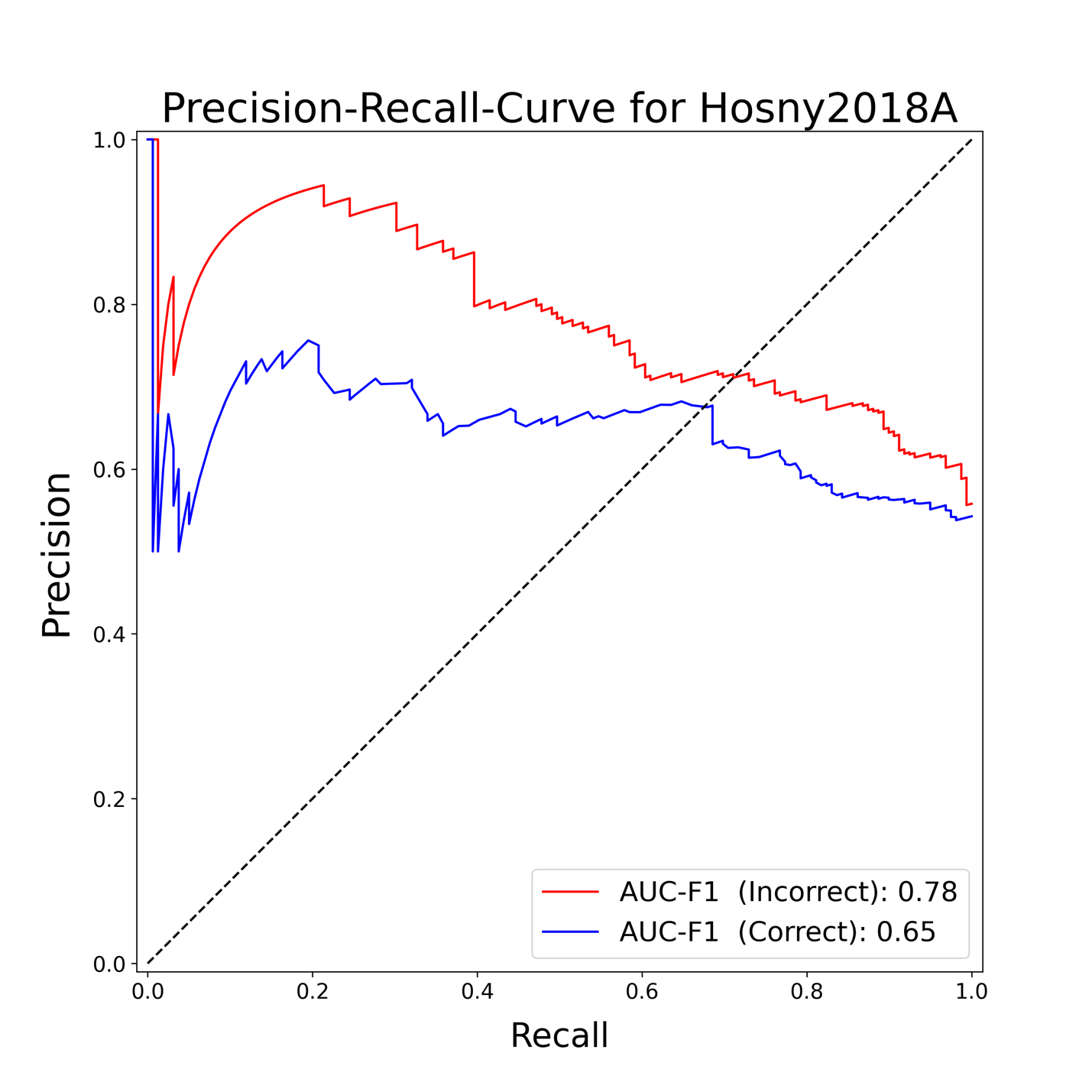

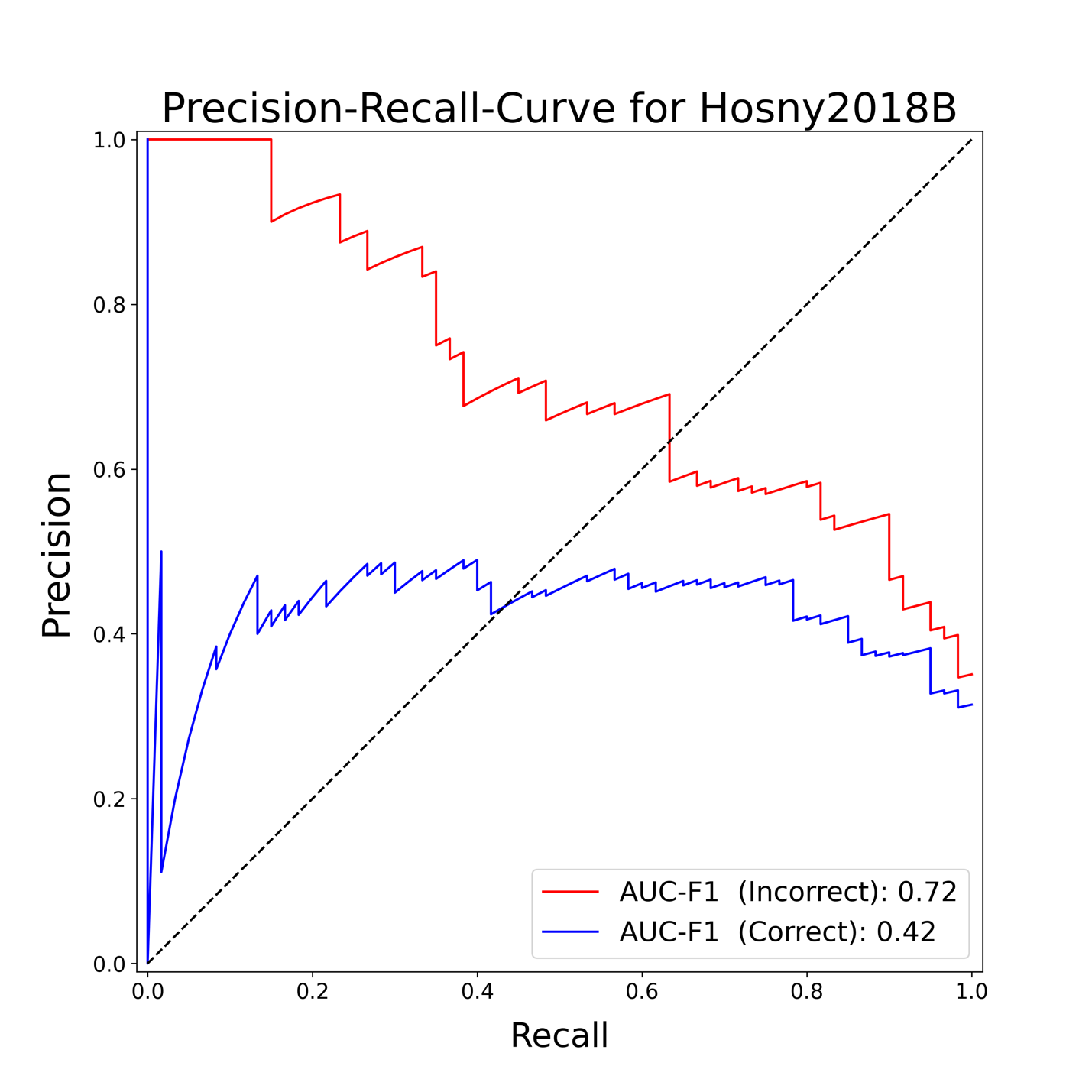

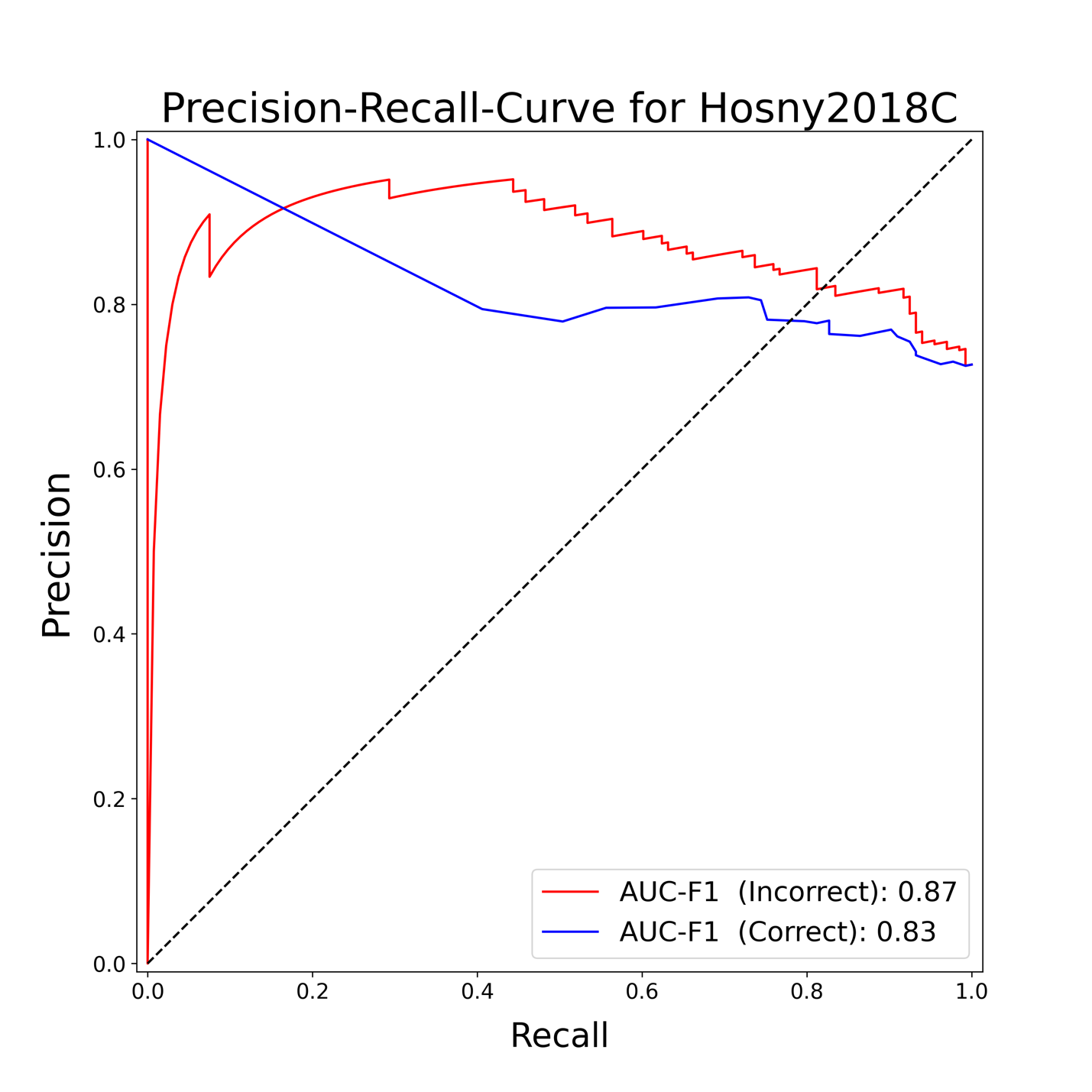

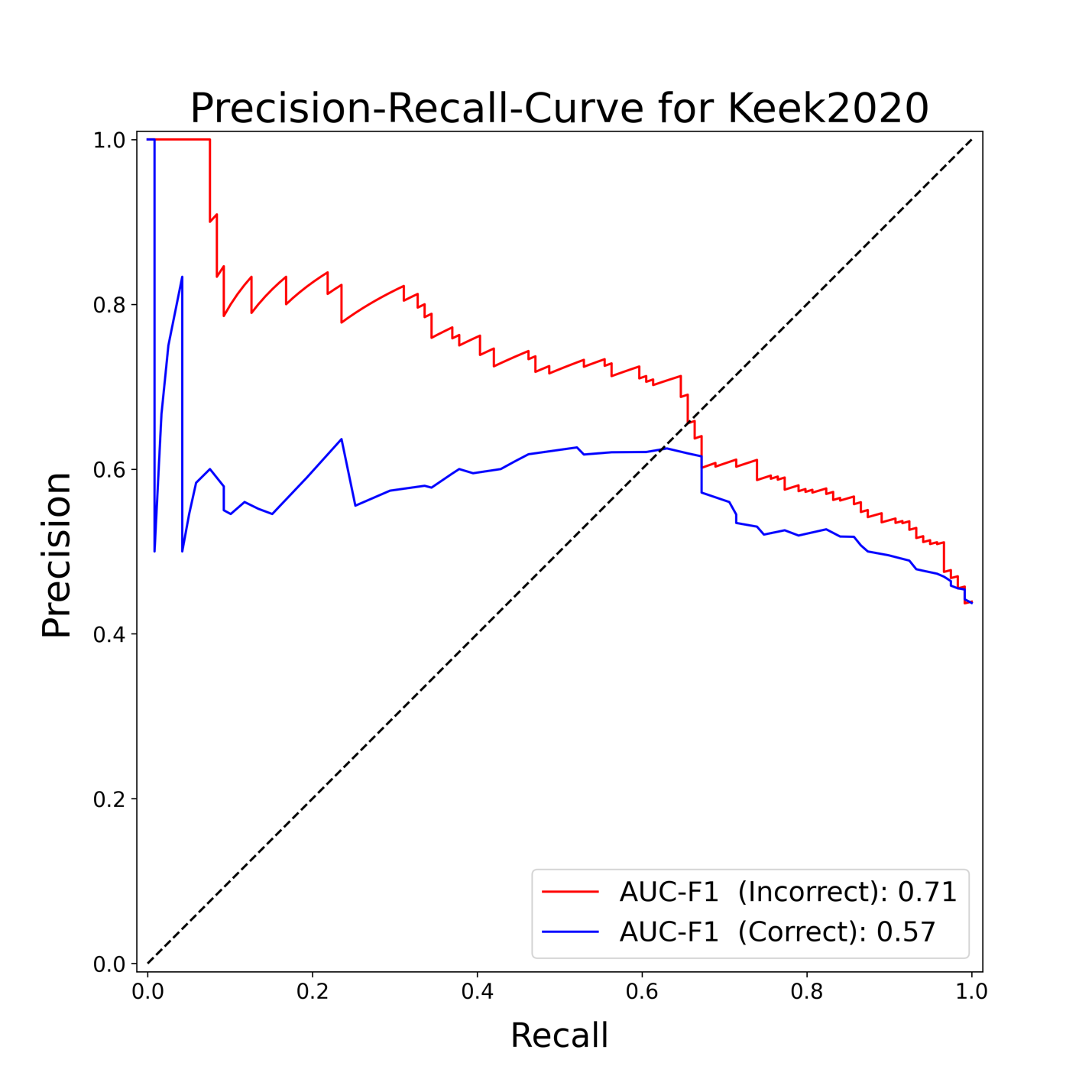

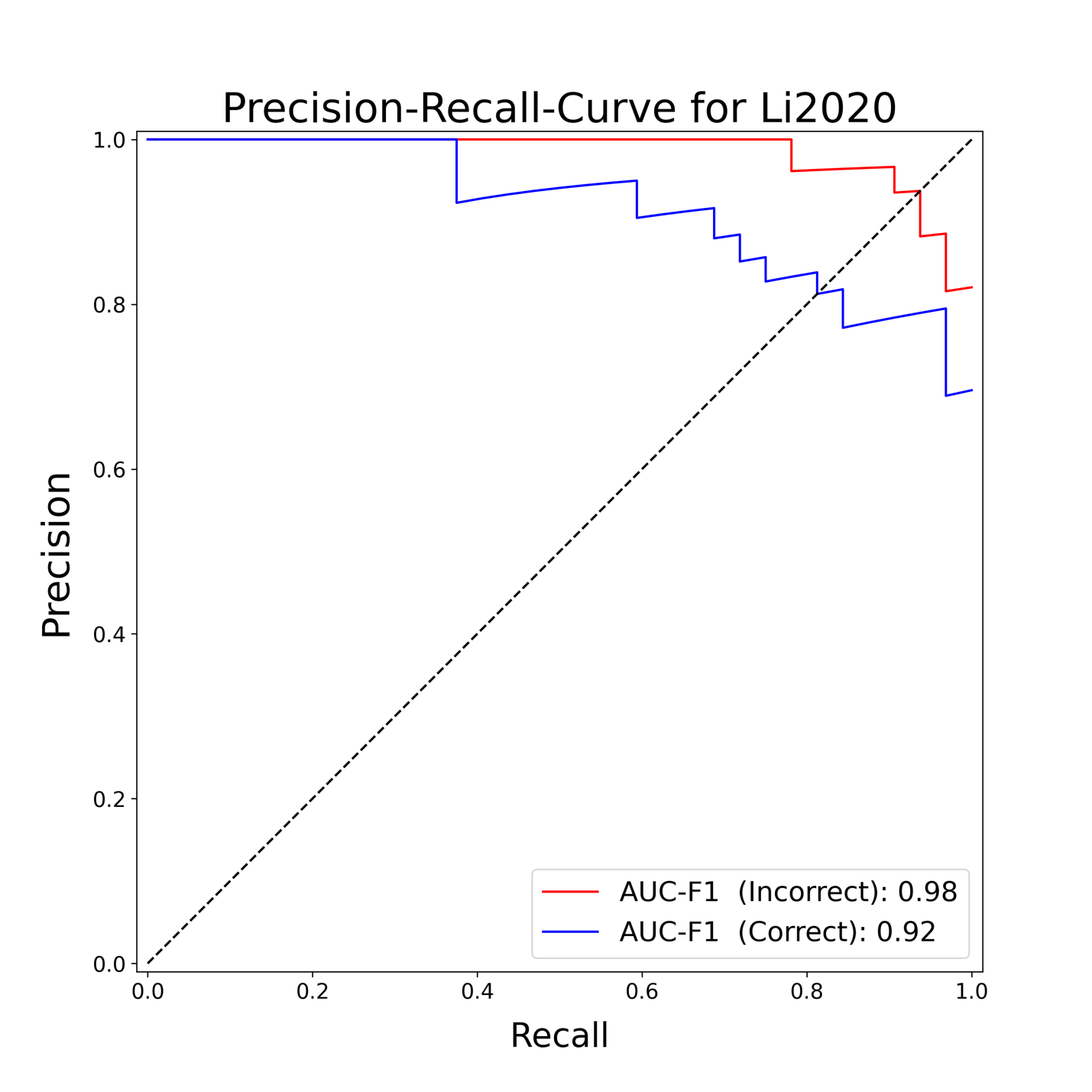

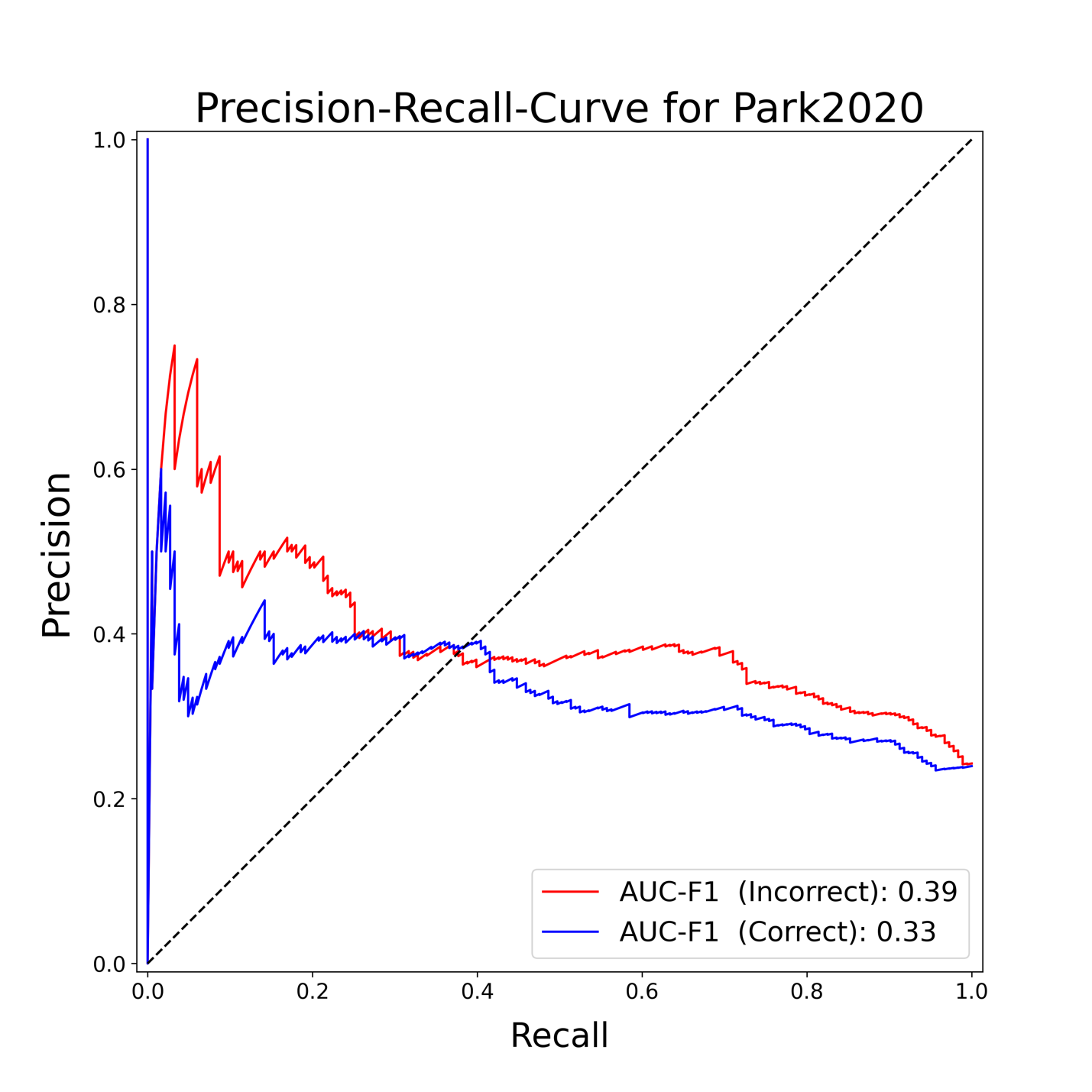

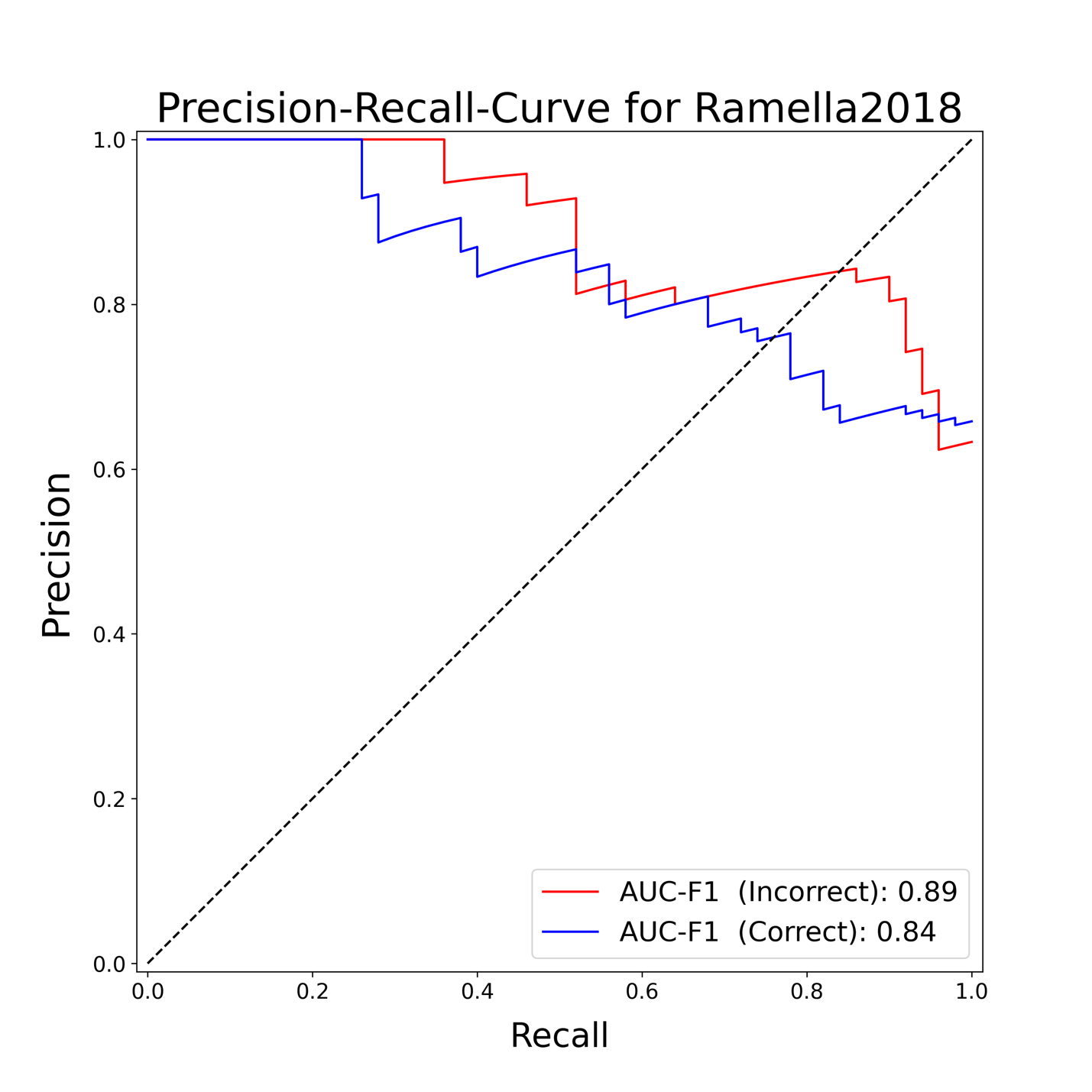

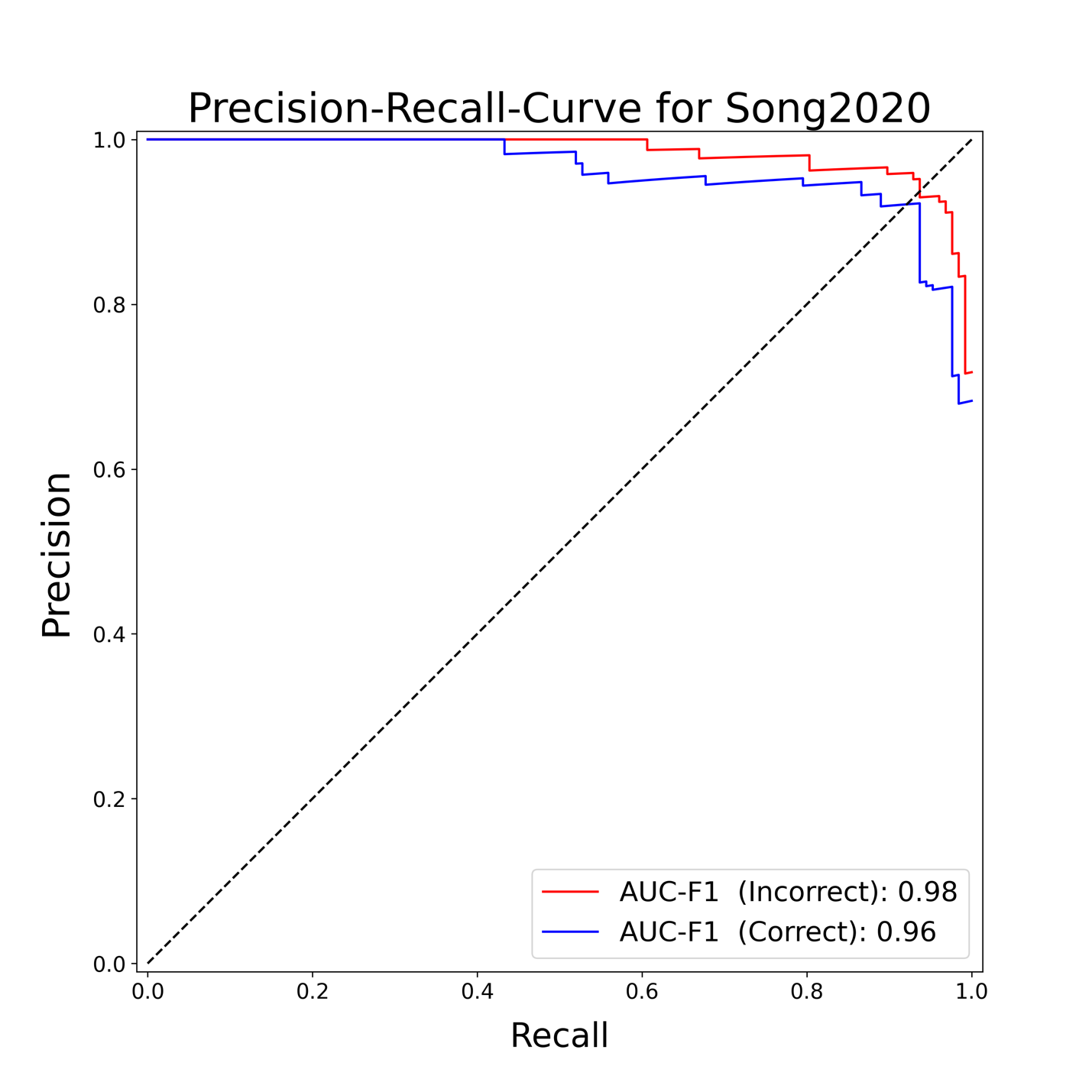

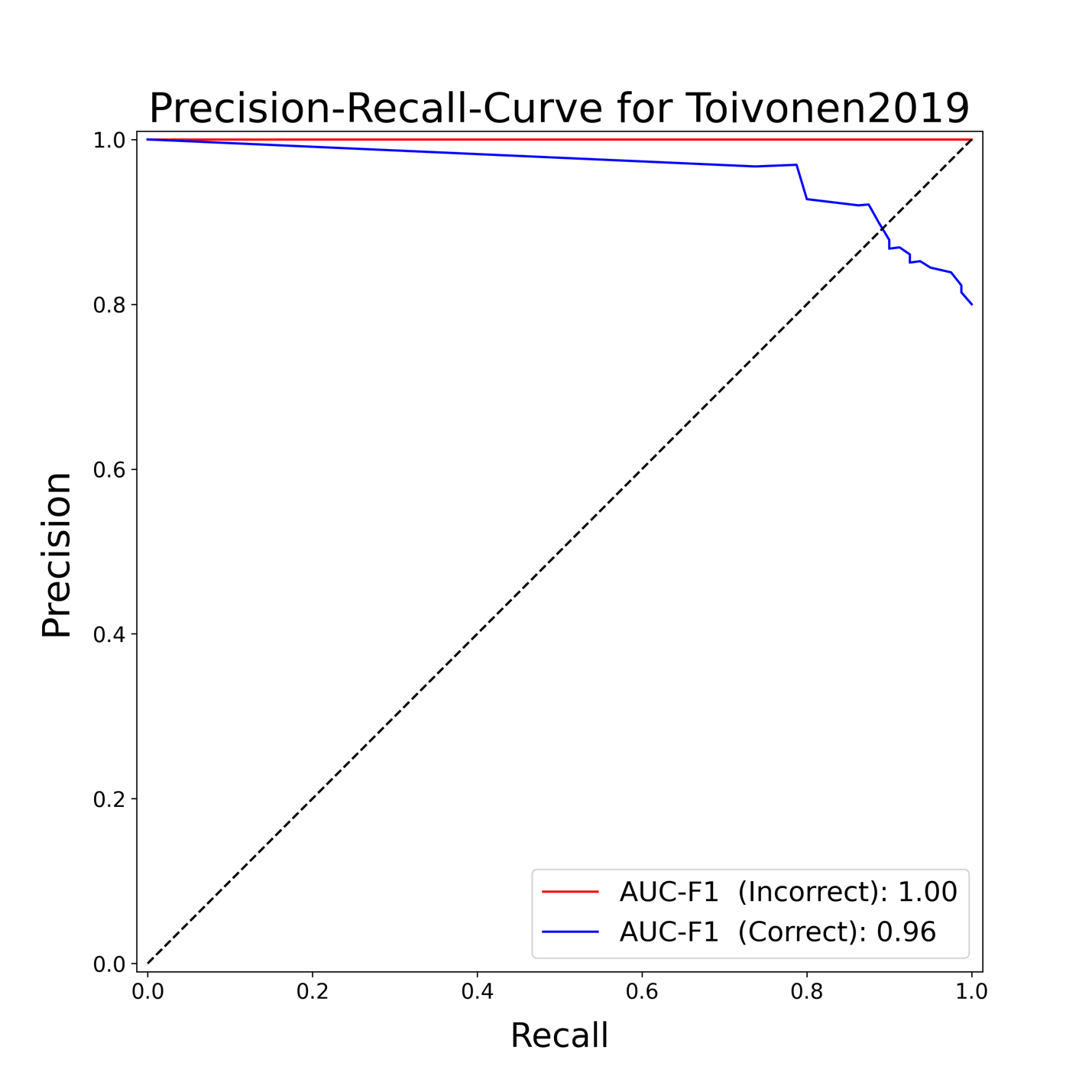
**

**Feature Selection/Classifier Importance**

**
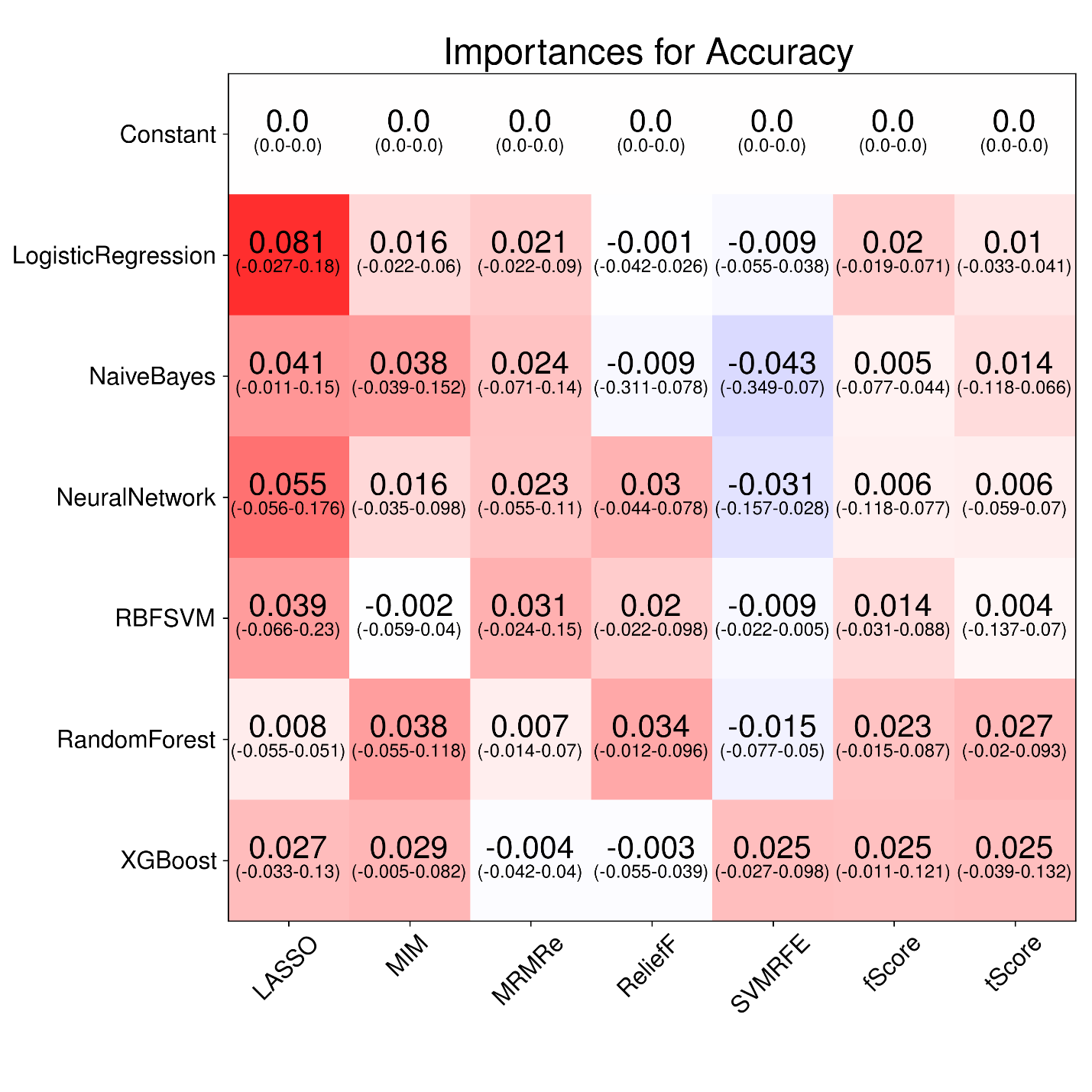

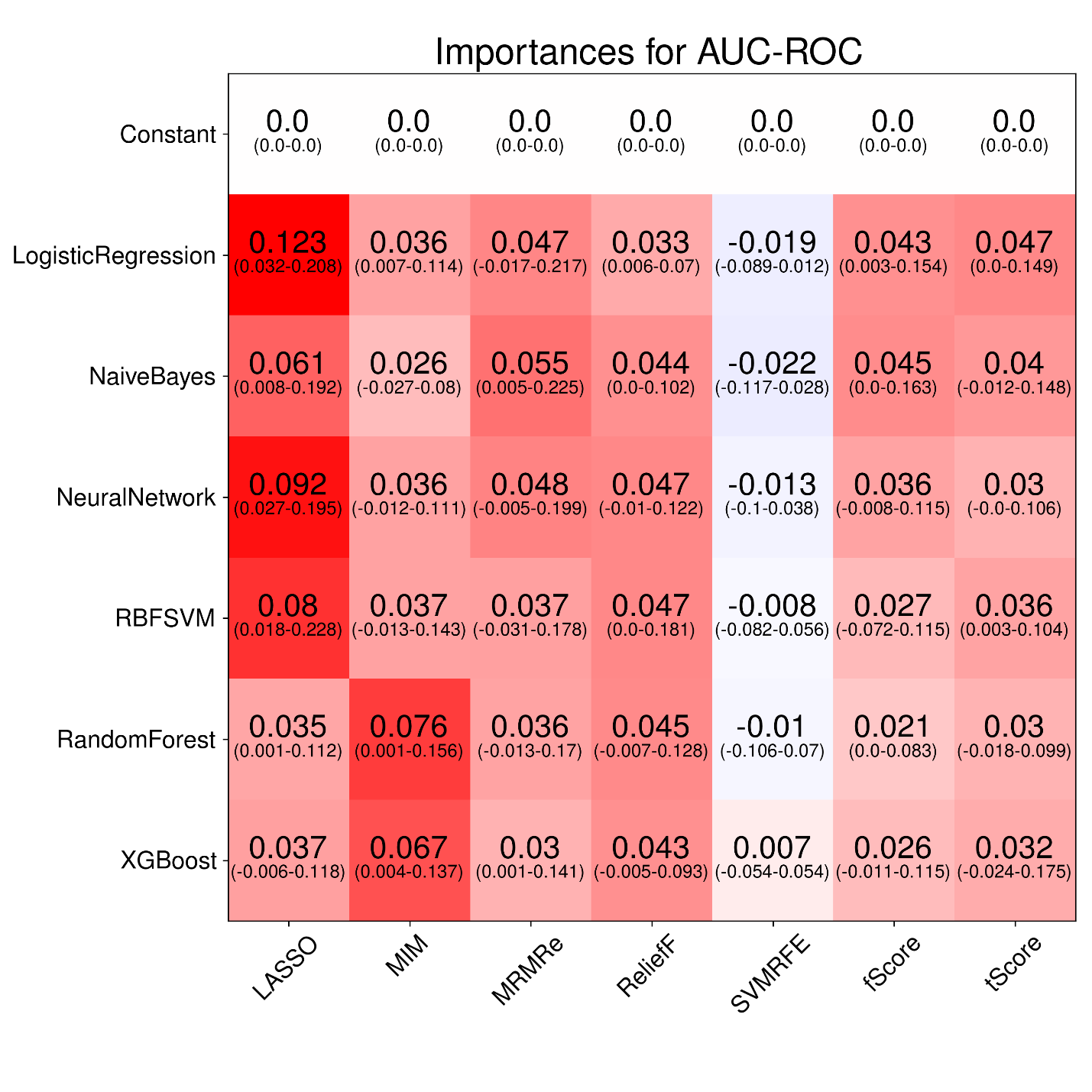

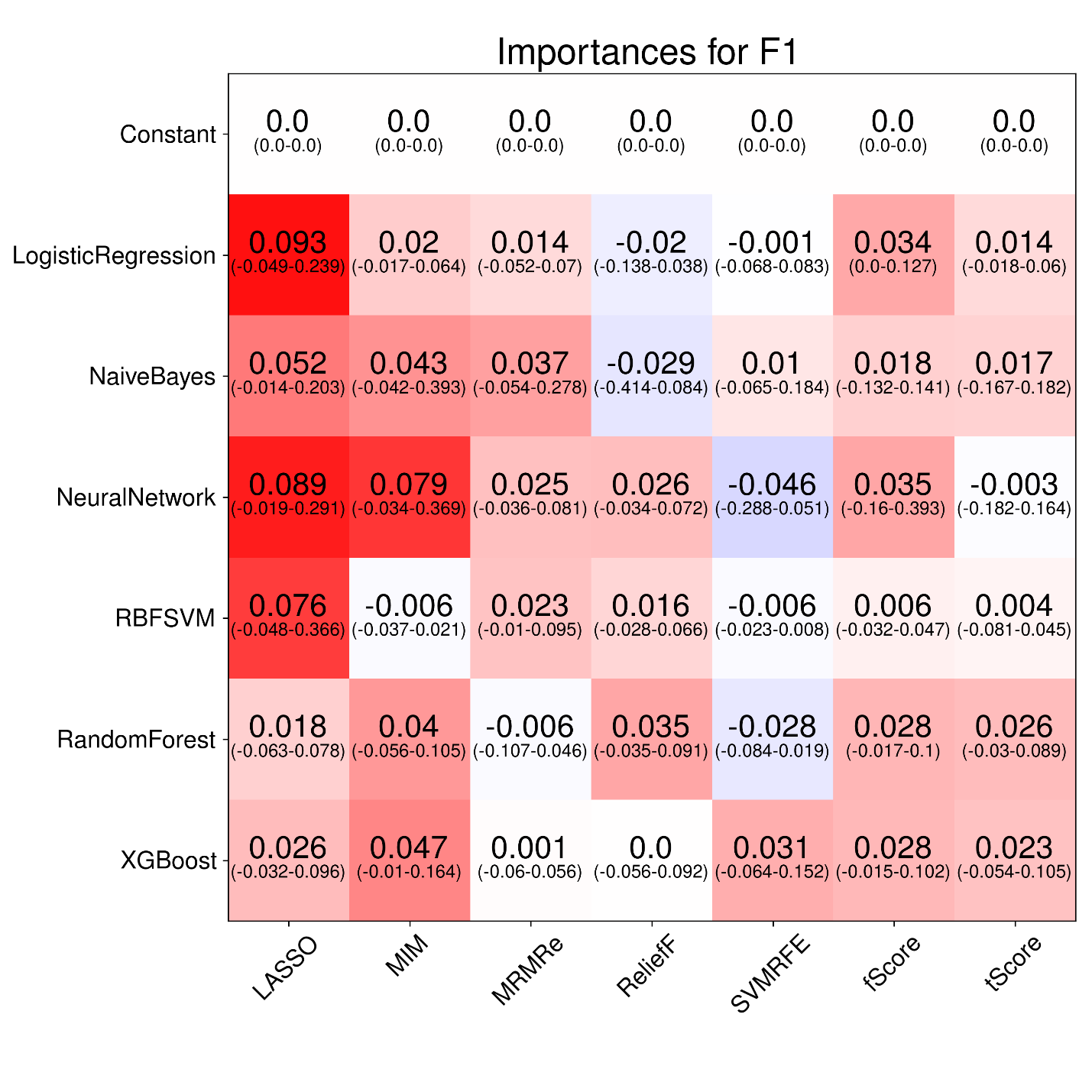

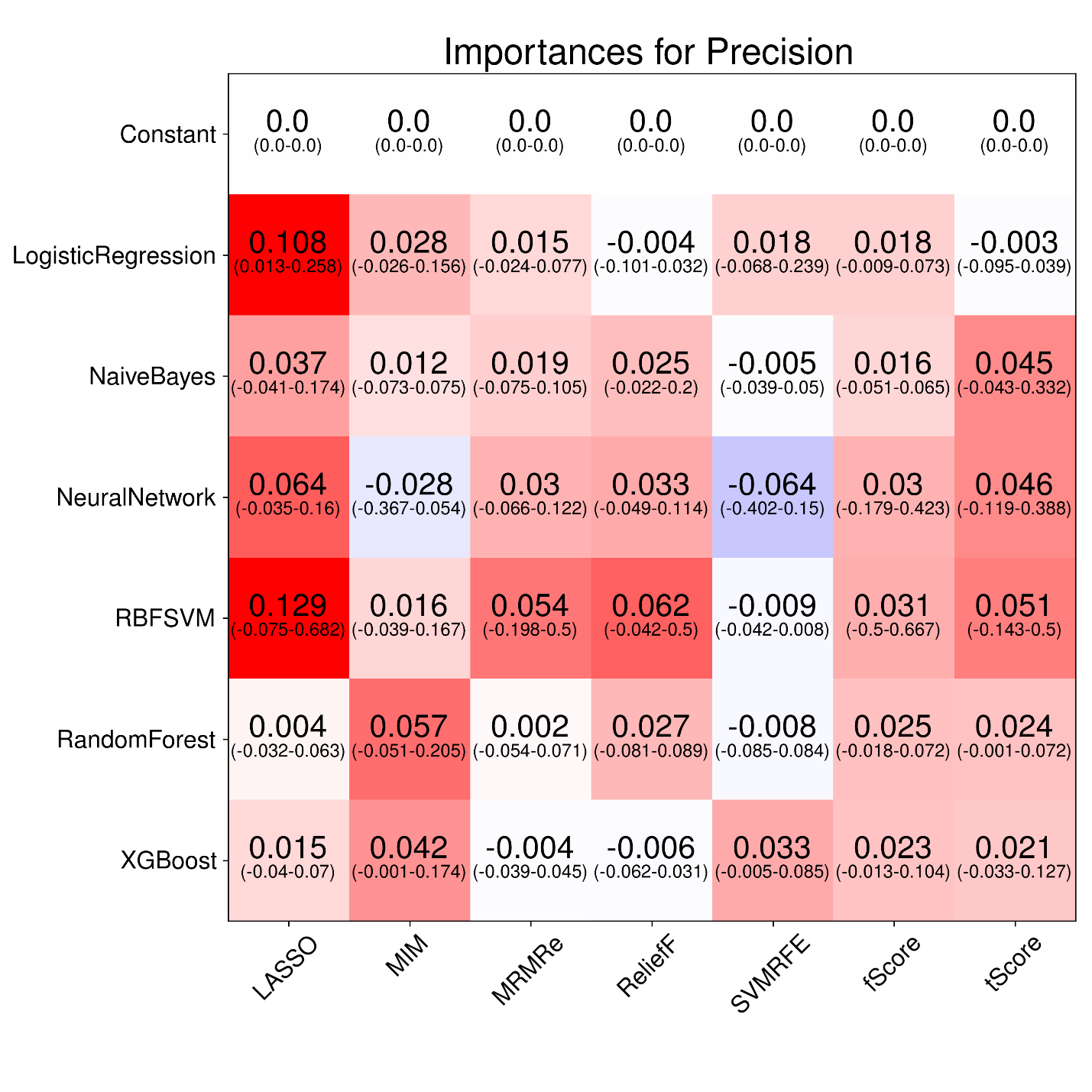

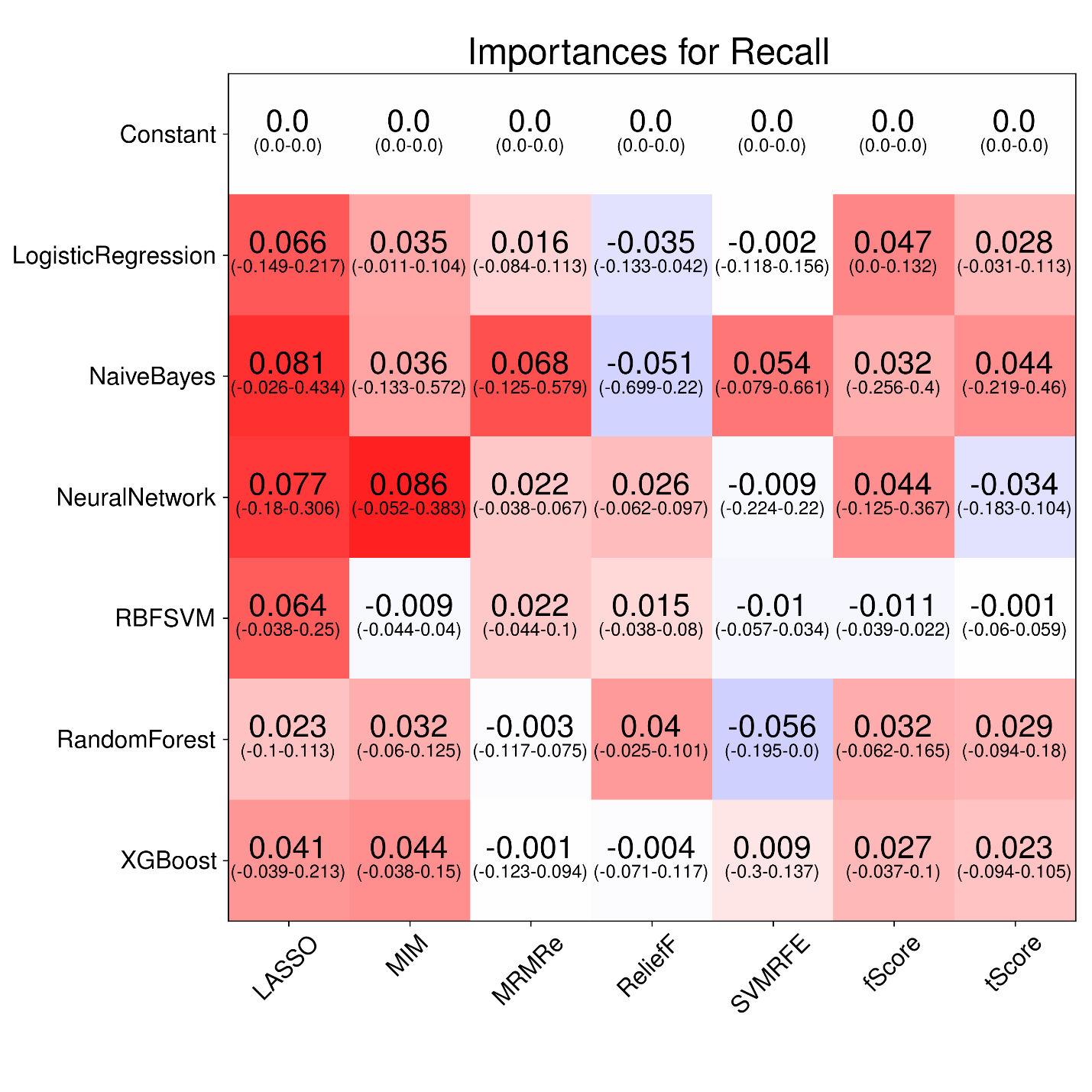

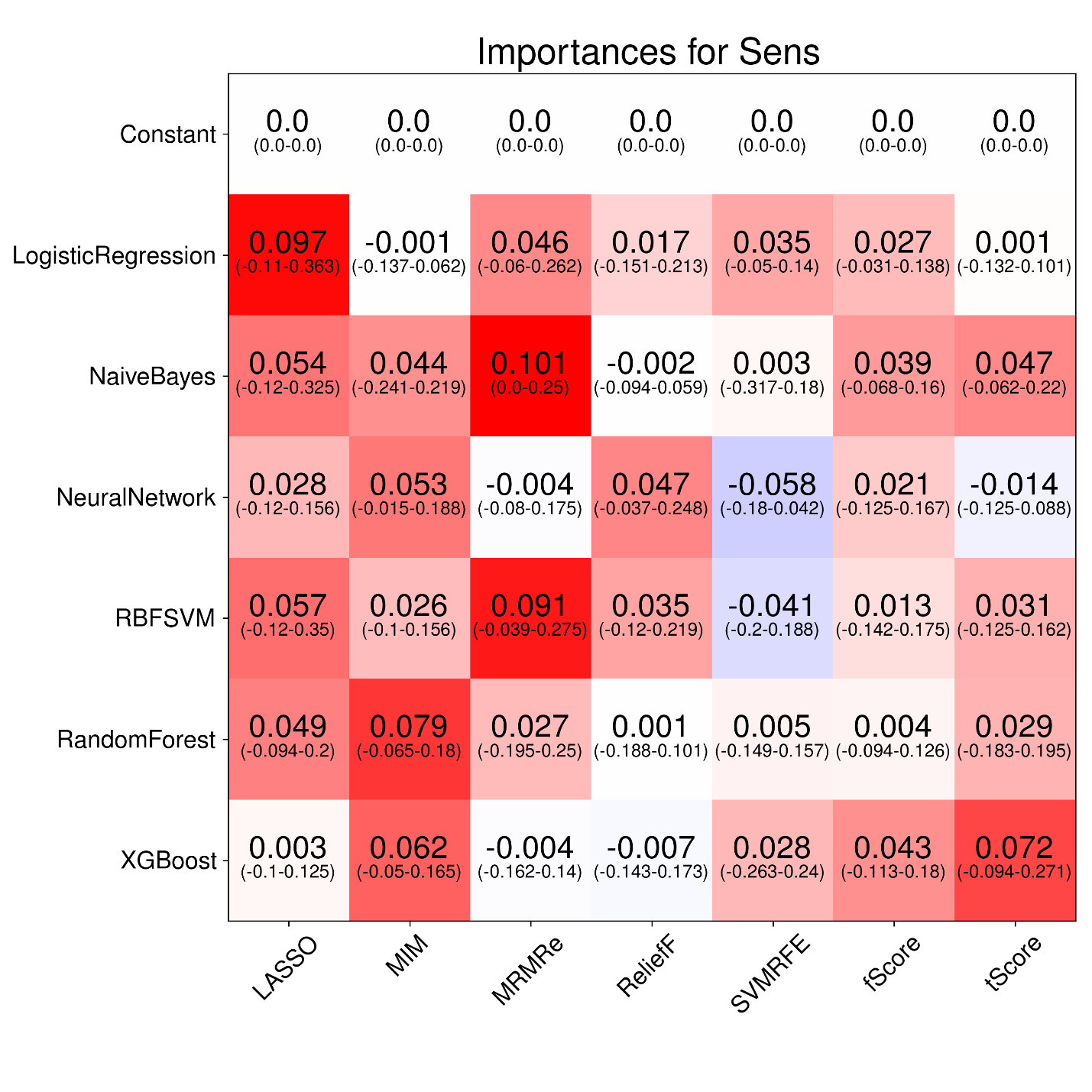

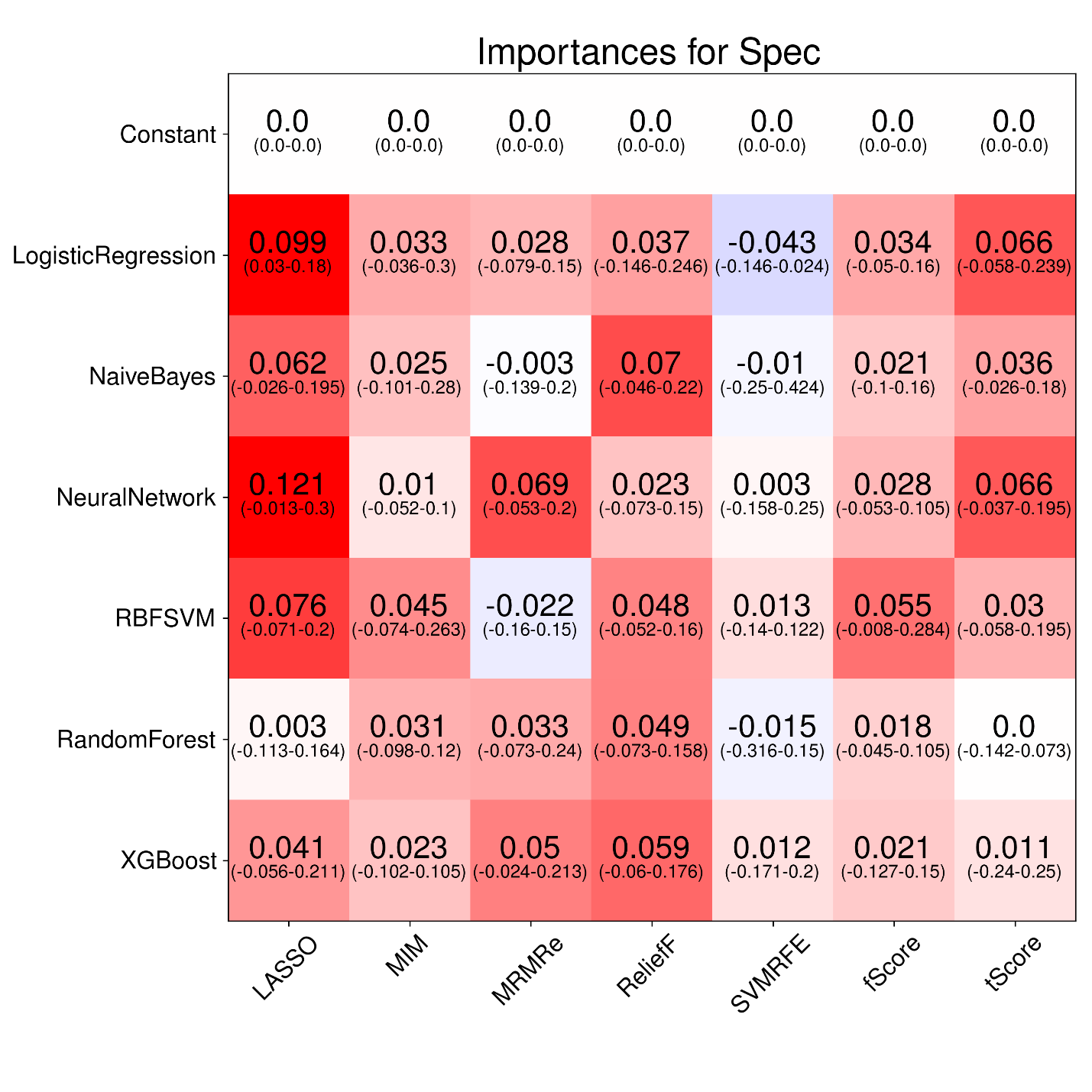
**

**Bias vs Dimensionality**

**
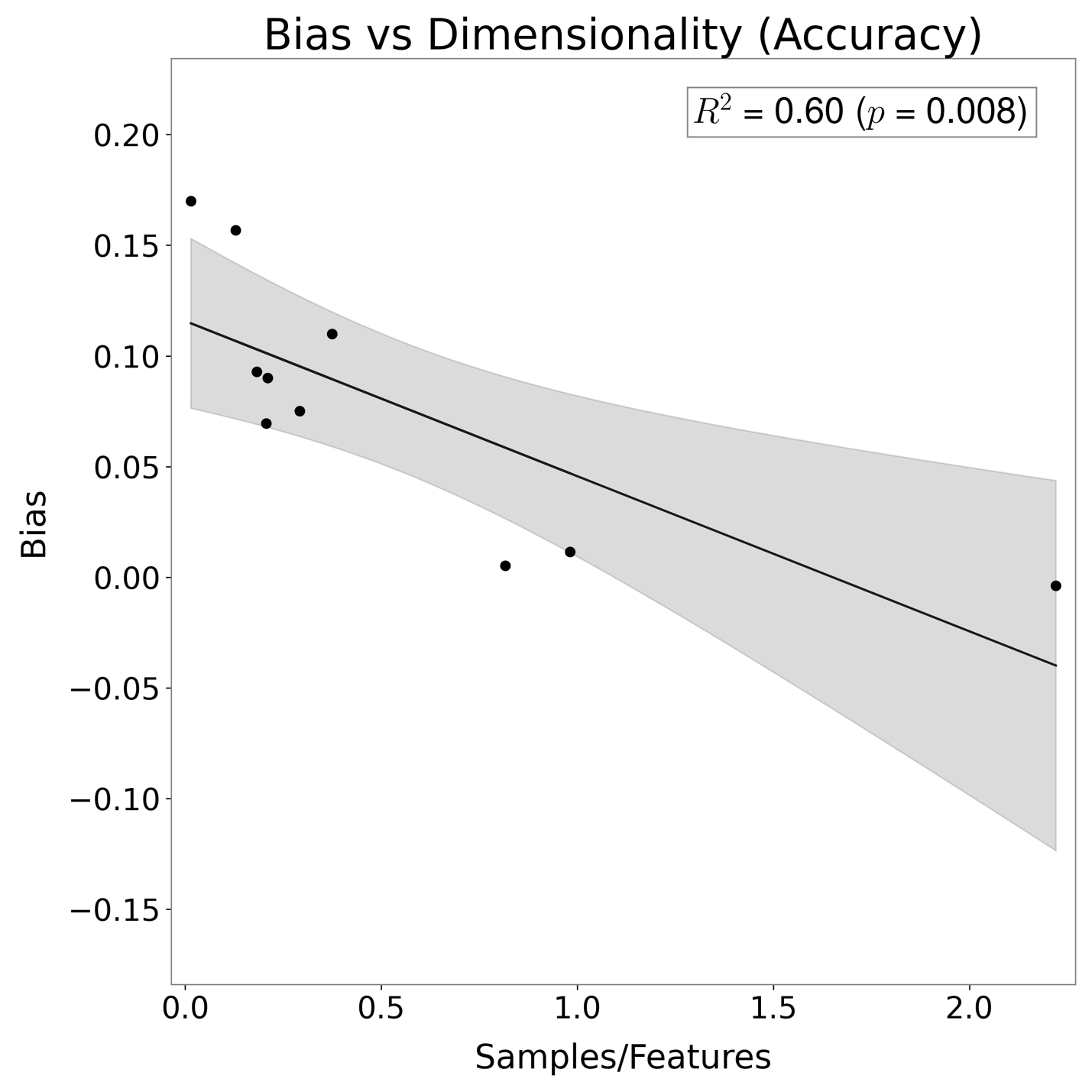

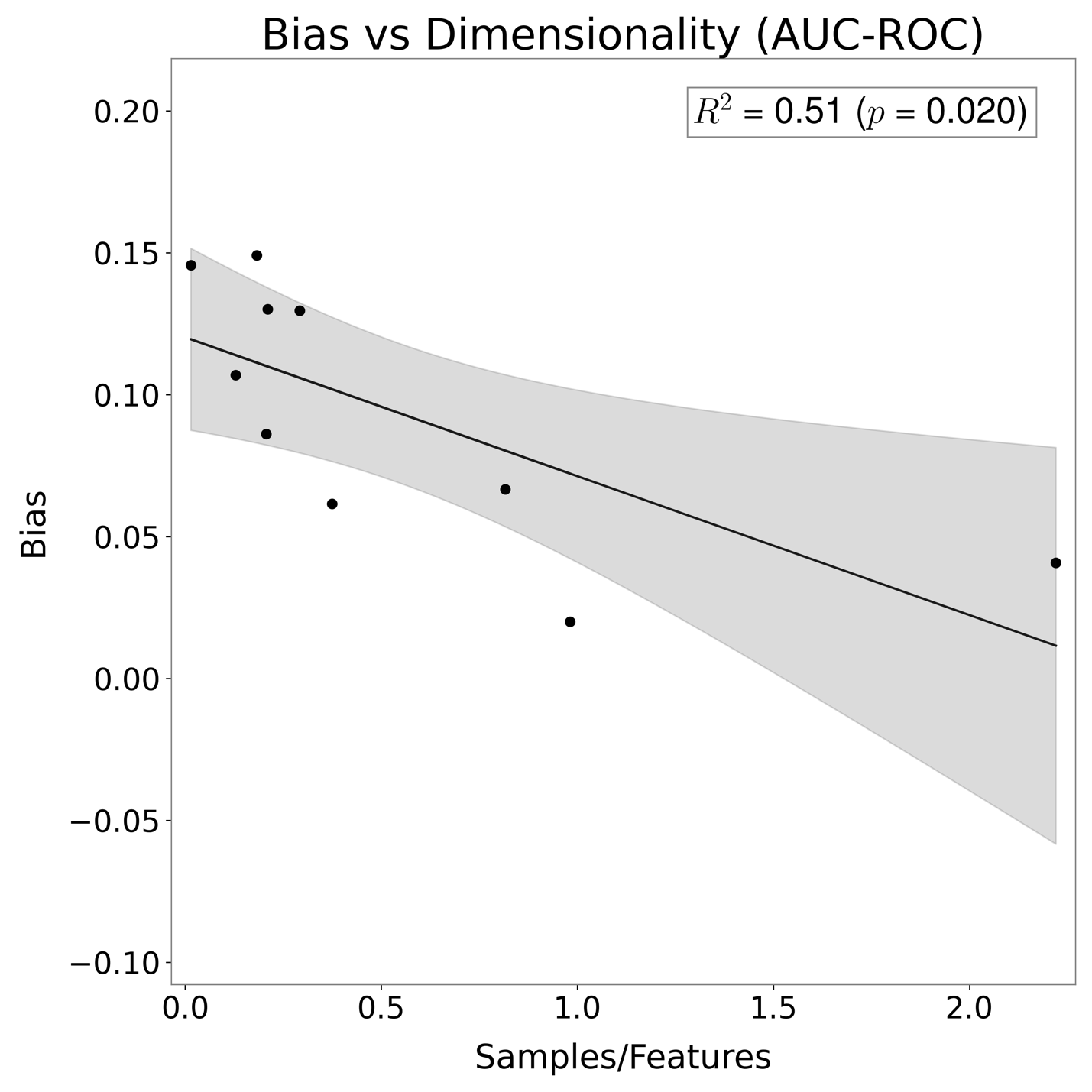

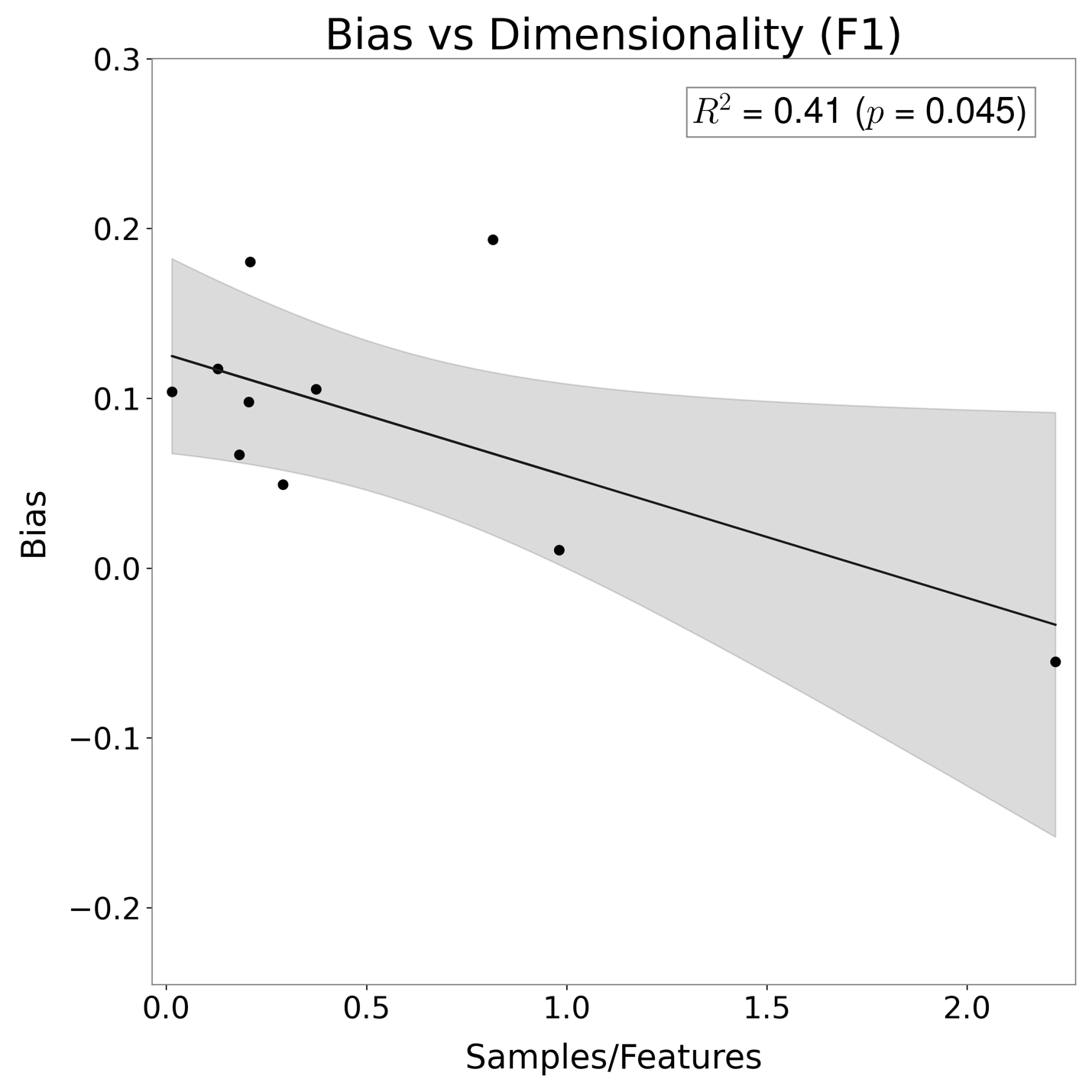

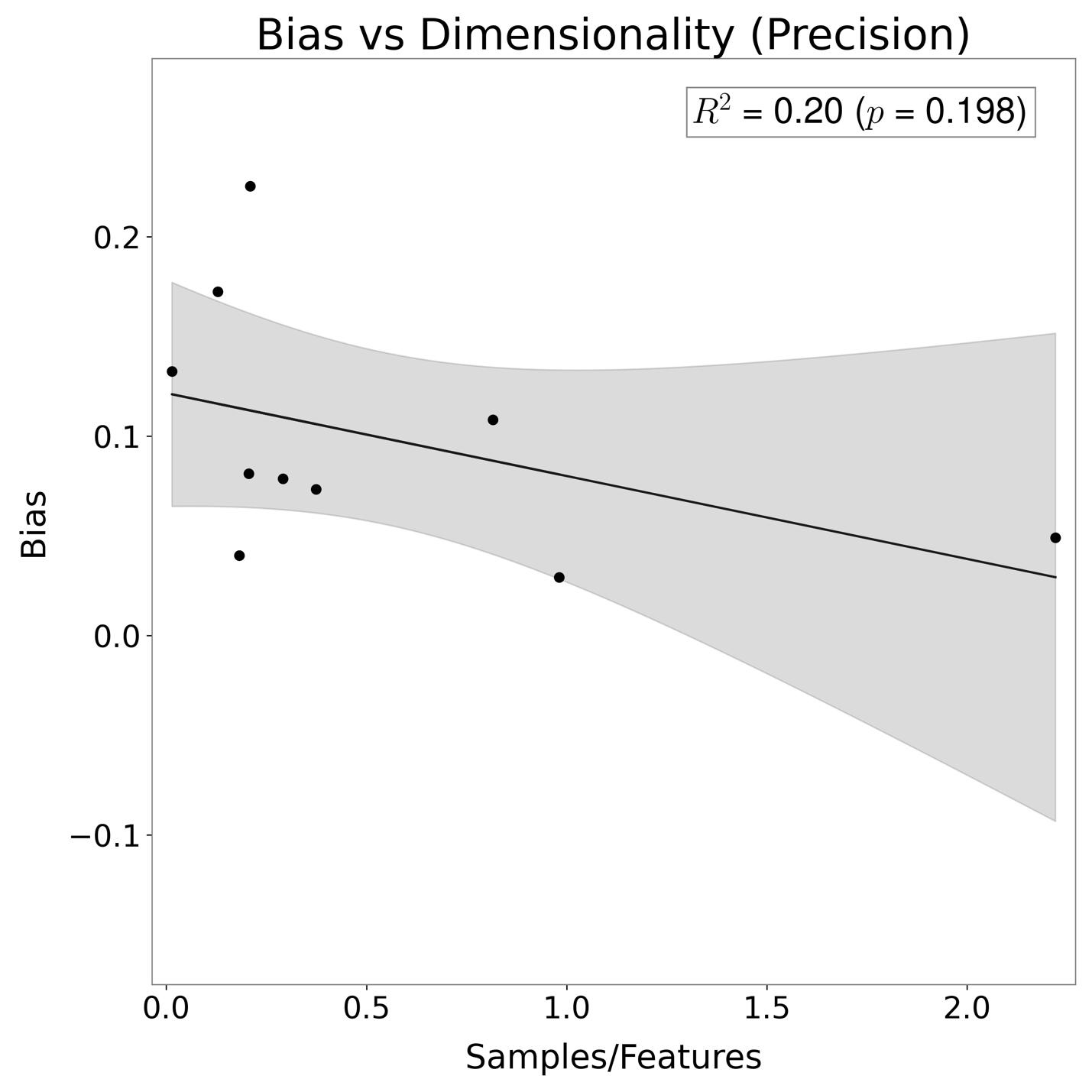

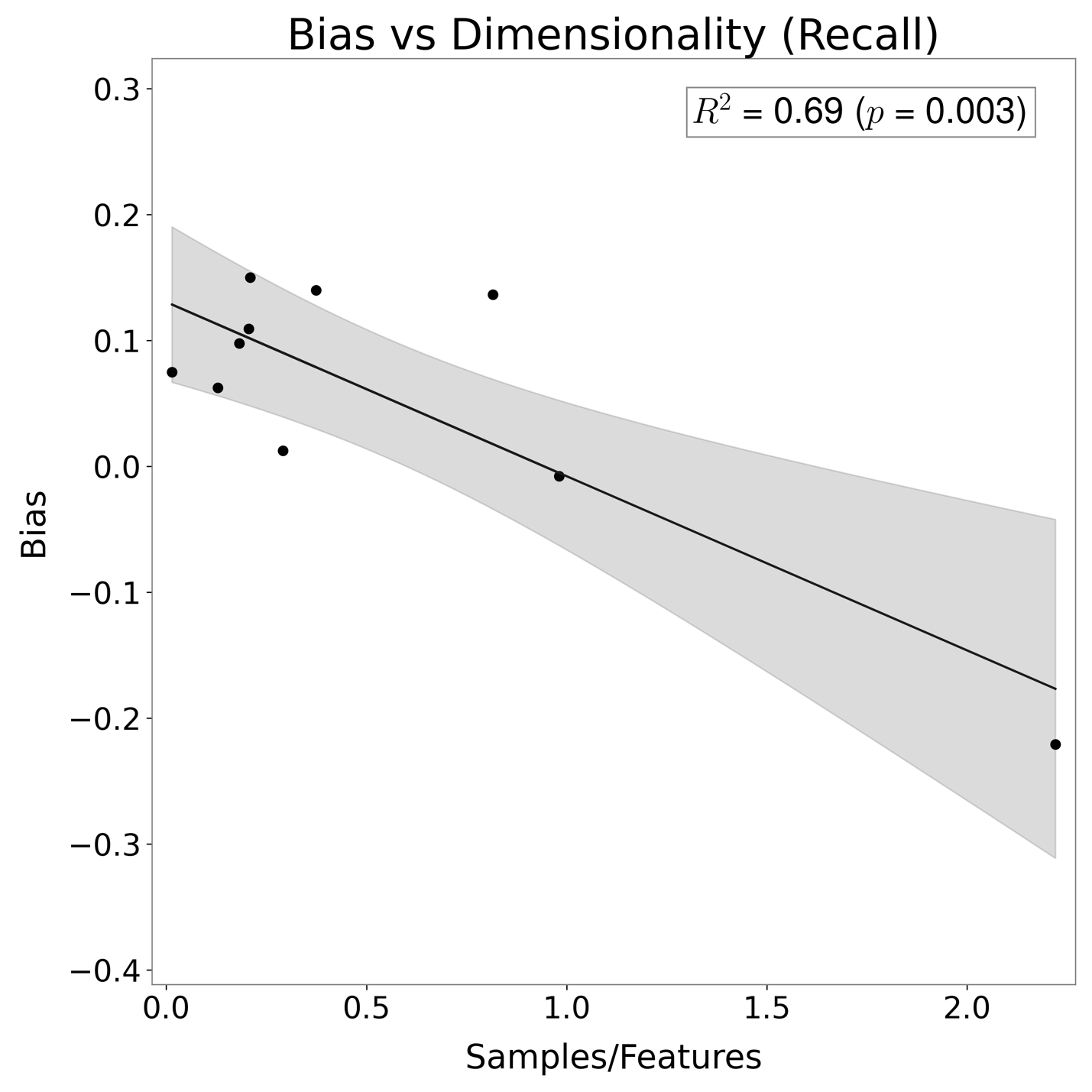

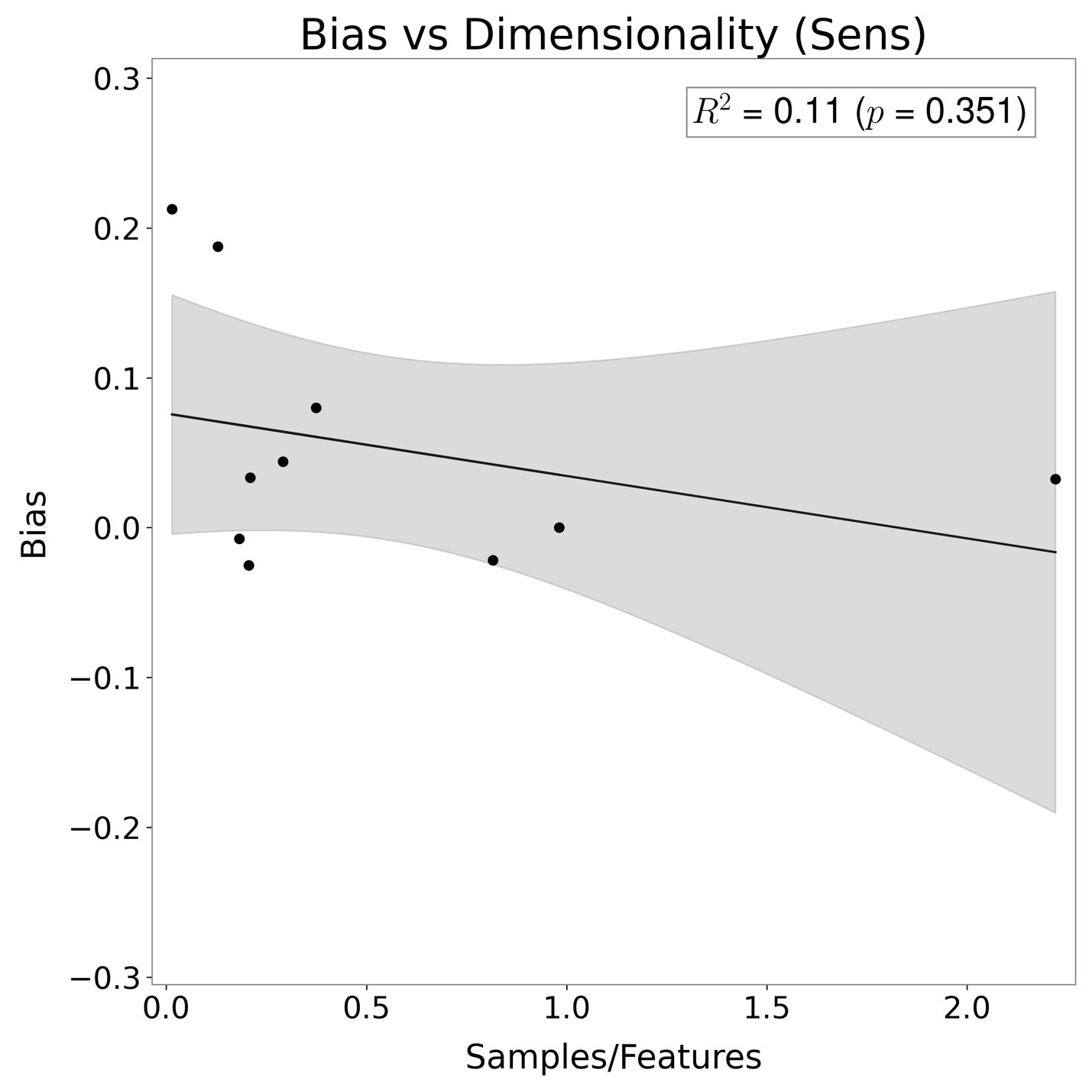

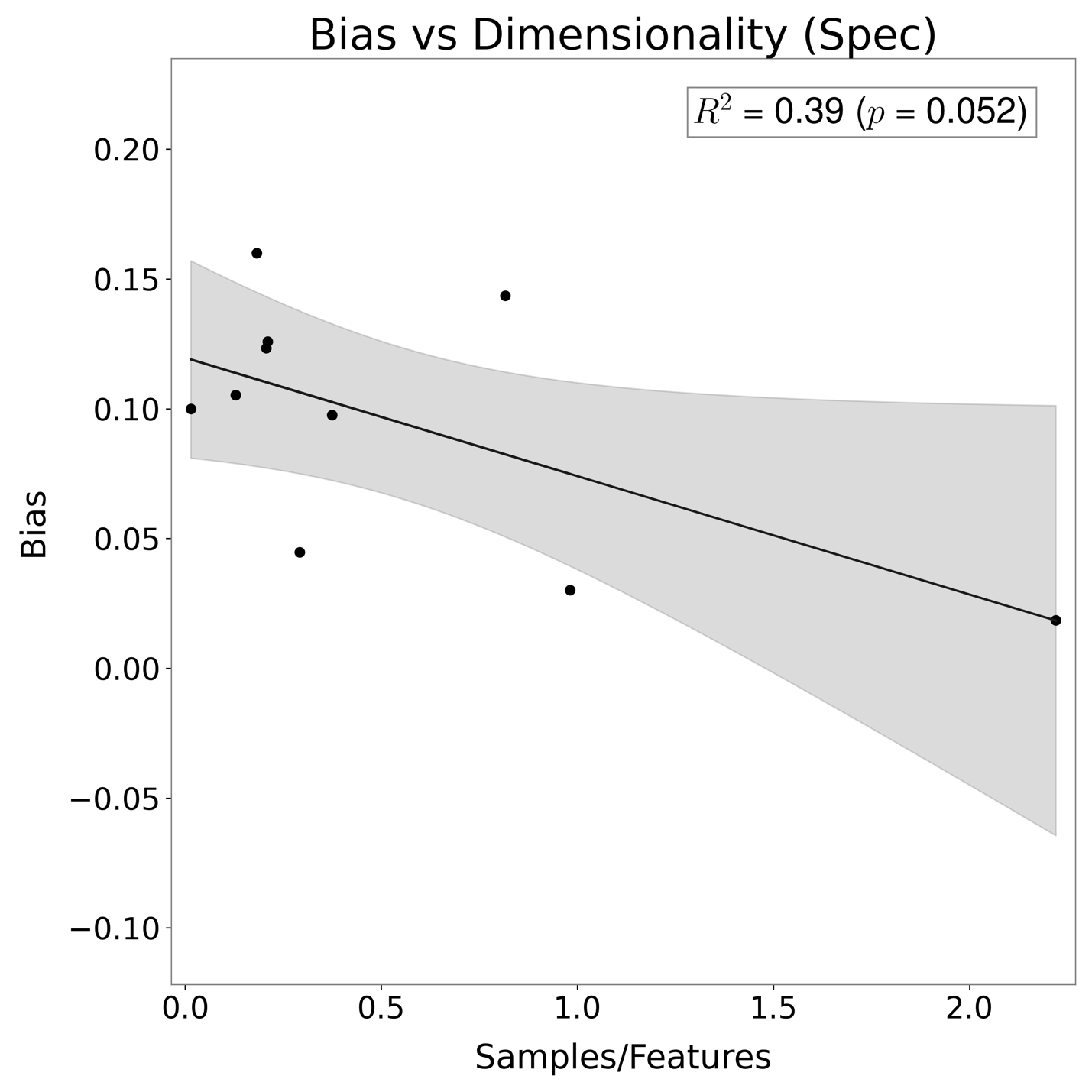
**
